# Synergistic melanoma cell death mediated by inhibition of both MCL1 and BCL2 in high-risk tumors driven by NF1/PTEN loss

**DOI:** 10.1038/s41388-021-01926-y

**Published:** 2021-07-30

**Authors:** Shuning He, Mark W. Zimmerman, Hillary M. Layden, Alla Berezovskaya, Julia Etchin, Megan W. Martel, Grace Thurston, Chang-Bin Jing, Ellen van Rooijen, Charles K. Kaufman, Scott J. Rodig, Leonard I. Zon, E. Elizabeth Patton, Marc R. Mansour, A. Thomas Look

**Affiliations:** 1 Department of Pediatric Oncology, Dana-Farber Cancer Institute, Harvard Medical School, Boston, MA USA; 2 Stem Cell Program and Division of Hematology/Oncology, Children’s Hospital Boston, Howard Hughes Medical Institute, Boston, MA USA; 3 Department of Pathology, Brigham and Women’s Hospital, Boston, MA USA; 4 MRC Human Genetics Unit, MRC Institute of Genetics and Molecular Medicine, University of Edinburgh, Edinburgh, UK; 5 Department of Hematology, UCL Cancer Institute, University College London, London, UK

**Keywords:** Cancer models, Melanoma

## Abstract

Melanomas driven by loss of the NF1 tumor suppressor have a high risk of treatment failure and effective therapies have not been developed. Here we show that loss-of-function mutations of *nf1* and *pten* result in aggressive melanomas in zebrafish, representing the first animal model of NF1-mutant melanomas harboring PTEN loss. MEK or PI3K inhibitors show little activity when given alone due to cross-talk between the pathways, and high toxicity when given together. The mTOR inhibitors, sirolimus, everolimus, and temsirolimus, were the most active single agents tested, potently induced tumor-suppressive autophagy, but not apoptosis. Because addition of the BCL2 inhibitor venetoclax resulted in compensatory upregulation of MCL1, we established a three-drug combination composed of sirolimus, venetoclax, and the MCL1 inhibitor S63845. This well-tolerated drug combination potently and synergistically induces apoptosis in both zebrafish and human NF1/PTEN-deficient melanoma cells, providing preclinical evidence justifying an early-stage clinical trial in patients with NF1/PTEN-deficient melanoma.

## Introduction

Cutaneous melanoma accounts for the vast majority of skin cancer-related deaths. More than 100,000 newly diagnosed cases of melanoma are projected in the United States for 2020 together with ∼6800 melanoma-related deaths [1]. The Cancer Genome Atlas (TCGA) classified cutaneous melanomas into four molecular subtypes: *BRAF*-mutant (47.5%), *RAS*-mutant (29%), *NF1*-mutant (9%), and triple-wild type (14.5%) [2]. The *NF1*-mutant category refers to cases lacking either *BRAF* or *RAS* mutations, whereas *NF1* mutations can also arise as a mechanism of resistance to RAF/MEK-targeted therapies in *BRAF*-mutated melanoma [3, 4]. Thus, *NF1* mutations have been reported in 13–17% of cutaneous melanomas overall [2, 5, 6].

The *NF1* gene encodes neurofibromin, a 2818-amino-acid protein whose GTPase-activating protein-related domain negatively regulates RAS signaling by catalyzing the hydrolysis of RAS-GTP into RAS-GDP. Thus, one consequence of *NF1*-loss is the aberrant activation of RAS signaling [7]. In primary melanoma patient biopsies, *NF1* mutations were not correlated with hot-spot *BRAF* mutations, a finding consistent with a redundant role for these two types of mutations in activating RAS-MAPK signaling.

Recent efforts to develop improved targeted therapies for melanoma have mainly focused on the *BRAF*-mutant subtype, leaving a paucity of treatment options for patients with *NF1*-mutant melanomas. It is unlikely that the FDA-approved BRAF-mutant-specific inhibitors will be beneficial against *BRAF*-wild-type, *NF1*-mutant melanomas. Moreover, analysis of multiple clinical trials indicate that the *NF1*-mutant subtype has the worst outcome among all metastatic melanomas [8]. Clearly, a better understanding of the molecular pathogenesis of *NF1*-mutant melanomas is needed to improve the design and hence the outcome of treatments for this subtype of melanoma.

A major impediment to the development of targeted therapies for patients with *NF1*-mutant melanomas has been the lack of suitable animal models. For example, both the *BRAF*-mutant and *RAS*-mutant subtypes of melanoma have been successfully modeled in mice [9] and zebrafish [10] by combining the melanoma-associated mutations in these genes with mutation or loss of *p53* or *Cdkn2a*, which are both typically inactivated in human melanoma [2, 11]. However, *Nf1*-loss was not sufficient to induce melanoma tumorigenesis in mice [3, 12] or zebrafish [13], either alone or in combination with *p53* loss. As the NF1-mutant melanomas often harbor a high mutation load [14, 15], we reasoned that genetic or epigenetic alterations affecting genes other than *p53* and *Cdkn2a* are likely required in combination with *NF1-*loss to initiate melanoma transformation in vivo. Because a significant subset of human *NF1*-mutant melanomas harbor genetic alterations leading to activation of the PI3K-AKT-mTOR pathway [2, 5], we hypothesized that targeting this pathway through inactivation of *ptena/ptenb* would drive melanomagenesis in *nf1/p53*-mutant zebrafish.

## Results

### Loss-of-function mutations of *nf1* and *pten* cooperate to drive melanomagenesis in *p53*-deficient zebrafish

**Fig. 1:**
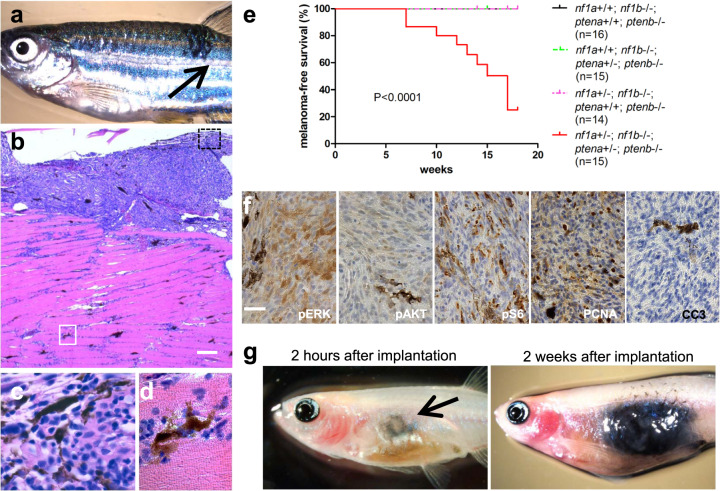
*nf1a*^*+/−*^*;nf1b*^*−/−*^*;ptena*^*+/−*^*;ptenb*^*−/−*^*;p53*^*M214K/M214K*^ zebrafish spontaneously develop melanomas with rapid growth. **a** Representative 16-week-old *nf1a*^*+/−*^*;nf1b*^*−/−*^*;ptena*^*+/−*^*;ptenb*^*−/−*^*;p53*^*M214K/M214K*^ zebrafish with one spontaneous melanoma (indicated by arrow). **b** Hematoxylin and eosin (H&E) staining of the melanoma tumor shown in panel a (×5 magnification, scale bar = 200 μm). **c** Melanoma tumor cells from the black box in (**b**), magnified ×100. **d** Melanoma tumor cells that have invaded into the dorsal muscle from the white box in (**b**), magnified ×100. **e** Cumulative frequency of spontaneous melanomas arising in zebrafish with the indicated genotypes (generated by the inbreeding of the *nf1a*^*+/−*^*;nf1b*^*−/−*^*;ptena*^*+/−*^*;ptenb*^*−/−*^*;p53*^*M214K/M214K*^ line, *p* < 0.0001, log-rank test). **f** Immunohistochemical analysis of melanoma tumor sections using antibodies to detect phosphorylated ERK1/2 (pERK), phosphorylated AKT (pAKT), phosphorylated S6 (pS6), proliferating cell nuclear antigen (PCNA) and cleaved caspase 3 (CC3) (×63 magnification, scale bar = 20 μm). The percentage of PCNA+ cells was determined by manually counting positive and negative melanoma cells in one representative high-power field (150–200 cells per field) within three independent tumor samples. **g** Pigmented *nf1/pten*-mutant melanoma cells were transplanted intraperitoneally into adult *rag2*^*−/−*^ Casper zebrafish. The implanted melanoma cells (left panel, arrow) grew rapidly into secondary tumors (within 2 weeks; right panel).

We previously reported the development of *nf1a*^*+/−*^*;nf1b*^−*/−*^ zebrafish lines with the loss of three of the four functional alleles of *nf1* [13]. These animals develop spontaneous malignant peripheral nerve sheath tumors (MPNSTs) with low penetrance, but not melanomas, beginning at the age of 1.5 years, indicating that *nf1*-loss alone is not sufficient to drive melanomagenesis (Supplementary Fig. S1). When we bred the *nf1a*^*+/−*^*;nf1b*^*−/−*^ line into a *p53*-deficient (*p53*^*M214K/M214K*^*)* background, the compound mutant fish developed MPNSTs or high-grade gliomas [13]. Although rare spontaneous melanomas were also detected, they had a very low penetrance (<2%) over the course of 40 weeks (Supplementary Fig. S1). Because human *NF1*-mutant melanomas often harbor gain-of-function alterations in the PI3K signaling pathway, including mutational inactivation of PTEN or overexpression of AKT3 [2, 5], we introduced *pten* loss-of-function mutations into *nf1a*^*+/−*^*;nf1b*^*−/−*^*;p53*^*M214K/M214K*^ zebrafish by crossing with a previously established *ptena*^*+/−*^*;ptenb*^*−/−*^ line [16, 17]. We then incrossed *nf1a*^*+/−*^*;nf1b*^*+/−*^*;ptena*^*+/−*^*;ptenb*^*+/−*^*;p53*^*+/M214K*^ fish and monitored the offspring for spontaneous tumor development every 2 weeks starting at 5 weeks of age. Very aggressive melanotic tumors began to appear in these fish at 7 weeks of age, with a penetrance of 80% by 20 weeks (Fig. 1a–e). Histopathologic study of the melanotic tumors revealed a dense, cellular neoplasm in which a subset of the neoplastic cells produced pigment, with an overall histology pathognomonic of malignant melanoma (Fig. 1b–d). Thus, activation of the PI3K pathway appears to be a critical requirement for melanomas to develop, in this case in concert with the loss of *NF1* and *p53*.

Melanomas arising in the *nf1/pten/p53*-mutant background were highly invasive into underlying musculature (Fig. 1b–d), and developed much earlier than melanomas in either the *Tg(mitf:BRAF*^*V600E*^)*;p53*^*M214K/M214K*^ or *Tg(mitf:NRAS*^*Q61K*^*);p53*^*M214K/M214K*^ zebrafish [18–21]. MPNSTs and glioblastomas appear in the *nf1/p53* background after 30 weeks of age, while melanomas develop starting at 5 weeks of age in the *nf1/pten/p53* background and grow so rapidly that fish usually need to be sacrificed for humane reasons before 30 weeks of age, so melanomas are the only tumor-type observed in the *nf1/pten/p53* background. Importantly, these spontaneous melanomas arose exclusively in fish that were homozygous null for both *nf1b* and *ptenb*, heterozygous for *nf1a* and *ptena* mutant alleles, and either heterozygous or homozygous for *p53*^*M214K*^ (Figs. 1 and S2). DNA PCR from melanoma tumors and adjacent normal tissue showed that the wild-type allele of *nf1a* and *ptena* is retained by the tumor cells (Supplementary Fig. S3). The *nf1a*^*+/−*^*;nf1b*^*−/−*^*;ptena*^*+/−*^*;ptenb*^*−/−*^*;p53*^*M214K/M214K*^ tumors (designated “*nf1/pten*-mutant melanomas”) developed at random sites across the surface of the fish (Fig. 1a). The pigmented melanoma cells were highly invasive, infiltrating skeletal muscle adjacent to every tumor examined for histology (Fig. 1b–d). Hence, retention of only one allele of both *nf1* and *pten* in a p53-mutant background drives the development of highly invasive malignant melanoma in our zebrafish model.

### The *nf1/pten*-mutant melanomas lack *braf/nras* hot-spot mutations

Since 80% of human cutaneous melanomas harbor activating hot-spot mutations in either *BRAF* or *NRAS* (e.g., *BRAFV600, NRASG12*, or *NRASQ61)* [2], we examined the *nf1/pten*-mutant zebrafish melanomas for spontaneous mutations at equivalent sites in the zebrafish orthologues (Supplementary Fig. S4). Sequencing of eight tumors revealed only wild-type alleles of these two genes in each tumor (Supplementary Fig. S5). Hence, similar to the *NF1*-mutant class of human cutaneous melanomas [2], the loss of *nf1* is sufficient to provide RAS pathway activation, and zebrafish melanomas in this background do not contain *braf/nras* hot-spot mutations.

### *nf1/pten*-mutant melanomas exhibit aberrant activation of the RAS and PI3K pathways and are highly proliferative

Since NF1 and PTEN are well-established negative regulators of RAS and PI3K signaling [7, 22], respectively, we postulated that the *nf1/pten*-mutant melanomas would exhibit activation of effector pathways downstream of *RAS* and *PI3K*. Indeed, we detected high levels of phosphorylated ERK (pERK), phosphorylated AKT (pAKT), and phosphorylated S6 ribosomal protein (pS6, an mTOR downstream effector) by immunohistochemistry (IHC) in the *nf1/pten*-mutant melanomas (Fig. 1f), indicating hyperactivation of both RAS and PI3K pathways. Because these pathways drive proliferation, we next analyzed the proliferative capacity of *nf1/pten*-mutant melanomas, observing high levels of expression of proliferating cell nuclear antigen (PCNA) in 45% of tumor-cell nuclei but not the adjacent normal tissue (Fig. 1f), indicating a high tumor proliferative rate. Apoptotic cells were not observed in these melanomas, as indicated by the lack of detectable cleaved caspase-3 (Fig. 1f). Hence, combined activation of the RAS and PI3K pathways, a high proliferative rate, and the lack of apoptosis likely account for the rapid onset and high growth rate of *nf1/pten*-mutant melanomas.

### *nf1/pten*-mutant melanomas can be serially transplanted into immunodeficient recipients

To assess the transplantation potential of our melanoma model, we isolated *nf1/pten*-mutant melanoma cells and transplanted them intraperitoneally into the optically clear immunodeficient *rag2*^*E450fs*^*(casper)* zebrafish [23] (designated “*rag2*^*−/−*^”). Robust engraftment was observed at the site of injection. All recipient fish demonstrated rapidly growing melanotic tumor masses within 2 weeks (Fig. 1g). Because of the invasive properties of the primary *nf1/pten*-mutant melanomas (Fig. 1), we also tested the feasibility of their intramuscular transplantation into *rag2*^*−/−*^ zebrafish, where the tumor cells not only expanded within muscle, but also invaded neighboring tissues such as the ventral fin (Supplementary Fig. S6). By contrast, non-transformed melanocytes (derived from normal pigmented melanocytes within the skin stripes) from *nf1a*^*+/−*^*;nf1b*^*−/−*^*;ptena*^*+/−*^*;ptenb*^*−/−*^*;p53*^*M214K/M214K*^ zebrafish failed to engraft in *rag2*^*−/−*^ zebrafish. Furthermore, although the melanomas arising from the *Tg(mitf:BRAF*^*V600E*^)*;p53*^*M214K/M214K*^ zebrafish [24] can be serially transplanted into *rag2*^*−/−*^ zebrafish, their post-transplantation growth rates were much slower, highlighting the extraordinarily high growth rate in vivo of the *nf1/pten*-mutant melanomas.

### MEK and PI3K inhibitors lack efficacy against *nf1/pten*-mutant melanomas in vivo

**Fig. 2:**
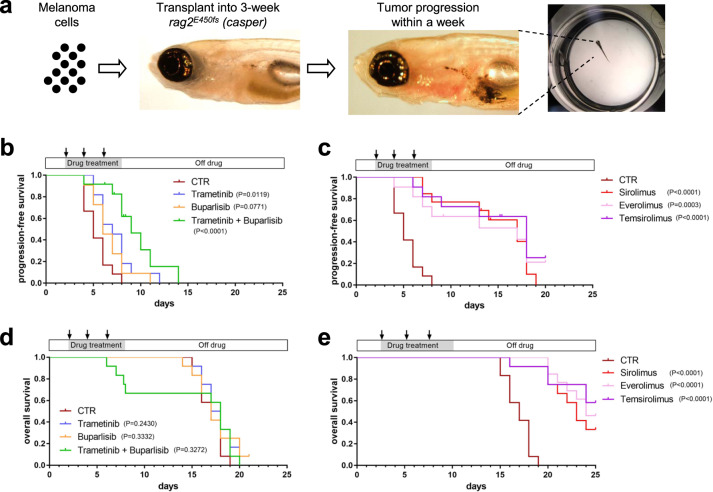
mTOR inhibitors achieve a durable antitumor effect in *nf1/pten*-mutant melanoma. **a** Schematic of the melanoma tumor transplantation assay. **b**, **c** Transplanted *nf1/pten*-mutant melanoma tumor cells were monitored daily in 3-week-old *rag2*^*−/−*^ recipient zebrafish treated with DMSO (CTR; *n* = 12), 80 nM trametinib (*n* = 11), 2 μM buparlisib (*n* = 11), or the combination of 80 nM trametinib and 2 μM buparlisib (*n* = 12) for 6 days. Kaplan–Meier curves for progression-free survival (PFS, **b**) and overall survival (OS, **c**) are shown. Statistical analyses were performed by log-rank test, comparing drug-treated with DMSO-treated zebrafish. **d**, **e** Transplanted *nf1/pten*-mutant melanoma tumor cells were monitored daily in 3-week-old *rag2*^*−/−*^ recipient zebrafish treated with DMSO (CTR; *n* = 12, same values as in **b**, **c**), 20 μM sirolimus (*n* = 12), 20 μM everolimus (*n* = 11) or 40 μM temsirolimus (*n* = 11) for 6 days. Kaplan–Meier curves are shown, with statistical analyses performed as in **b**, **c**. For all experiments involving drug treatments, drugs were replenished every 2 days during the 6-day course of treatment (black arrows).

Human *NF1*-mutant melanomas have the worst outcome among all metastatic melanomas [8], and *PTEN*-mutant melanomas are known to be resistant to T-cell mediated immunotherapy such as the immune checkpoint inhibitor [25]. Thus, there is a clear need for effective small-molecule inhibitors to overcome the aggressive growth properties of NF1/PTEN-mutant melanoma. Because targeting the RAS-MEK-ERK and PI3K-PTEN-AKT-mTOR signaling pathways might logically affect the growth of *nf1/pten*-mutant melanomas, we first transplanted these melanoma cells into 3-week-old *rag2*^*−/−*^ zebrafish and treated the recipients with MEK (trametinib or cobimetinb) or pan-PI3K (buparlisib or apitolisib) inhibitors. The *nf1/pten*-mutant melanoma cells grew rapidly in DMSO-treated recipients and progressed from an inoculum of 500 cells to readily detectable pigmented tumors at 4–8 days post transplantation (Fig. 2a). Single-agent treatment with either MEK or PI3K inhibitors from days 2–8 post transplantation at each of their maximum tolerated dosages (MTDs; Supplementary Fig. S7a) did not affect the growth of tumors (Figs. 2b and S7b). Even when tumor-bearing recipient fish were treated with a combination of trametinib and buparlisib at their MTDs (Supplementary Fig. S7a), tumor growth was only transiently inhibited during treatment, followed by rapid regrowth after drug removal, resulting in the lack of improvement in overall survival (Fig. 2d).

### Inhibition of mTOR suppresses the growth of *nf1/pten*-mutant melanomas in vivo

**Fig. 3:**
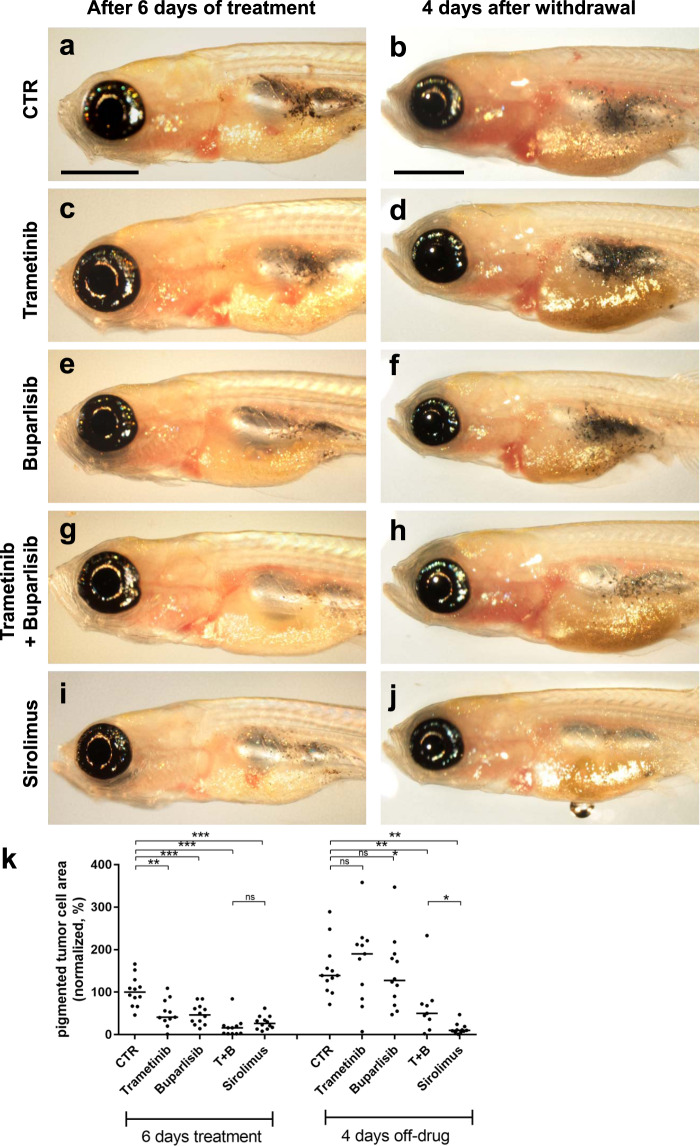
Sirolimus, but not trametinib or buparlisib, prevents rapid relapse of *nf1/pten*-mutant melanoma following treatment. Three-week-old *rag2*^*−/−*^ zebrafish transplanted with pigmented *nf1/pten*-mutant melanoma cells were treated for 6 days with DMSO, 80 nM trametinib, 2 μM buparlisib, the combination of 80 nM trametinib and 2 μM buparlisib, or 20 μM sirolimus. **a**, **c**, **e**, **g**, and **i** Representative zebrafish at the end of the 6-day drug treatment. **b**, **d**, **f**, **h**, and **j** Representative zebrafish at 4 days following the end of drug treatment. **k** Quantification of melanotic *nf1/pten*-mutant tumor-cell area at the end of the 6-day course of drug treatment (left), and 4 days later (right). ns *p* > 0.05, *p < 0.05, **p < 0.01, ***p < 0.001 by two-tailed, unpaired *t*-test. Scale bar = 1 mm.

To broaden the coverage of candidate pathway inhibitors, we next tested a panel of antitumor drugs targeting the RAS-MEK-ERK and receptor tyrosine kinase-PI3K-AKT-mTOR pathways in our *nf1/pten*-mutant melanoma model by assessing tumor-cell growth and overall survival of recipient *rag2*^−/−^ fish after 6 days of treatment (Figs. 2, S8 and S9). Among the 14 tested drugs, each at their MTD, only the rapamycin family of mTOR inhibitors (rapalogs) showed selective activity against *nf1/pten*-mutant melanoma in vivo as single agents. Interestingly, four different mTOR kinase inhibitors did not show activity against *nf1/pten*-mutant melanomas at their MTD (Supplementary Fig. S9). During the 6-day treatment course, sirolimus (rapamycin) clearly suppressed the appearance of detectable tumors, and its inhibition of tumor growth persisted for 1 to 2 weeks post treatment, in marked contrast to the rapid tumor regrowth in fish treated with MEK and PI3K inhibitors (Figs. 2c, e, 3 and S10). We also treated *nf1/pten*-mutant melanomas with everolimus and temsirolimus, two FDA-approved analogs of sirolimus. The three rapalogs showed similar abilities to durably inhibit melanoma cell growth (Fig. 2c, e), which uniformly translated to improved overall survival, indicating that rapalogs may provide a useful treatment option for these melanomas in vivo.

Primary *nf1/pten*-mutant tumors are invariably melanotic, but after serial transplantation, the tumor cells often become amelanotic [26]. In order to track the melanoma cells using EGFP instead of melanin, we bred the *sox10*:EGFP fluorescent zebrafish line into our *nf1/pten*-mutant line to aid in visualization of the transplanted melanoma cells, as they expressed high levels of the neural crest progenitor marker *sox10* (Supplementary Fig. S11) [24]. When transplanted into 3-week-old *rag2*^*−/−*^ zebrafish and treated for 6 days with multiple different inhibitors, the EGFP-expressing amelanotic cells responded poorly to single-agent treatment with either trametinib or buparlisib, had only temporary responses to the trametinib–buparlisib combination, but showed more durable responses to sirolimus and temsirolimus (Supplementary Fig. S12). Thus, the amelanotic melanoma cells appear to respond in a similar fashion to the melanotic melanoma cells, reinforcing the dependence of both subtypes of melanoma on mTOR signaling for malignant cell growth in vivo.

### Cell growth in *nf1/pten*-mutant melanomas depends on mTOR signaling

**Fig. 4:**
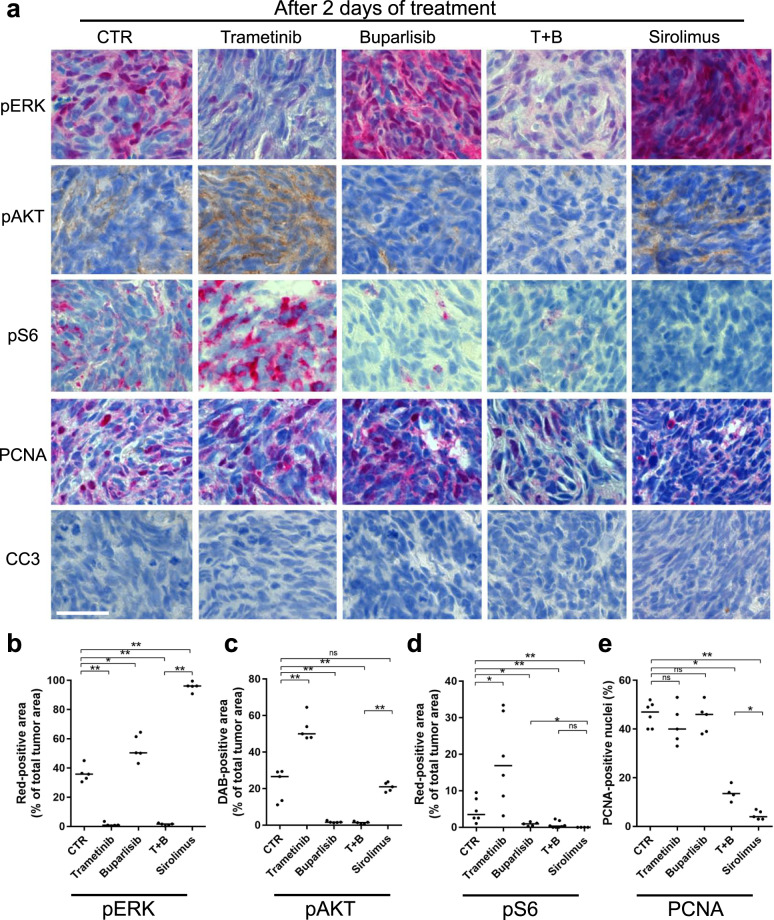
Sirolimus strongly inhibits proliferation in *nf1/pten*-mutant melanomas. **a** Representative tissue sections from a transplanted *nf1/pten*-mutant melanoma tumor after 2 days of treatment with DMSO (CTR), 80 nM trametinib, 2 μM buparlisib, the combination of 80 nM trametinib and 2 μM buparlisib, or 20 μM sirolimus. Sections were immunostained using antibodies to detect pERK, pAKT, pS6, PCNA, and cleaved caspase-3 (CC3). pERK-, pAKT- and pS6-positive tumor areas, as well as PCNA-positive nuclei, are quantified post-treatment in (**b**–**e**). “T + B” refers to trametinib plus buparlisib. ns *p* > 0.05, *p < 0.05, **p < 0.01 by Mann–Whitney test. Scale bar = 20 μm.

**Fig. 5:**
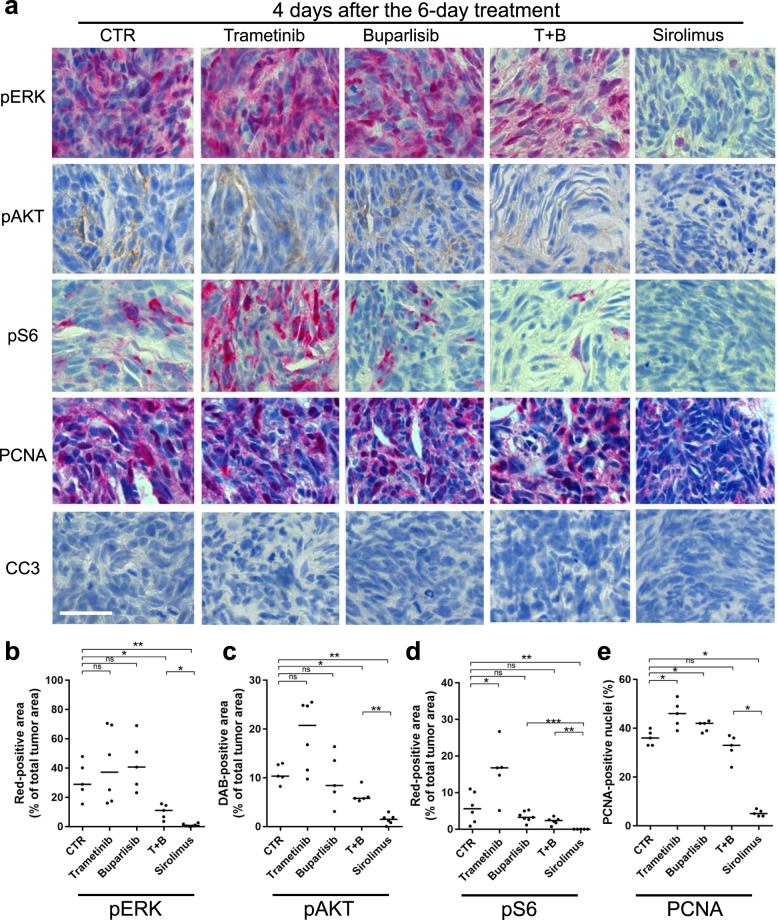
Sirolimus induces a durable cytostatic effect in *nf1/pten*-mutant melanomas. **a** Representative tissue sections from a transplanted *nf1/pten*-mutant melanoma tumor at 4 days after a 6-day drug treatment with DMSO (CTR), 80 nM trametinib, 2 μM buparlisib, the combination of 80 nM trametinib and 2 μM buparlisib, or 20 μM sirolimus. Sections were immunostained using antibodies to detect pERK, pAKT, pS6, PCNA, and CC3. pERK-, pAKT- and pS6-positive tumor areas, as well as PCNA-positive nuclei, are quantified in (**b**–**e**). “T + B” refers to trametinib plus buparlisib. ns *p* > 0.05, *p < 0.05, **p < 0.01, ***p < 0.0001 by Mann–Whitney test. Scale bar = 20 μm.

The RAS-MEK-MAPK and PI3K-AKT-mTOR pathways negatively regulate each other, such that a drug-induced blockade of one pathway results in increased activity of the other [27, 28]. To test whether these drugs act on the expected pathways in inhibitor-treated *nf1/pten*-mutant melanomas, we analyzed treated tumors by IHC, observing that treatment with the MEK inhibitor trametinib leads to a reduction in pERK levels (Fig. 4a, b), as expected; while levels of pAKT and pS6 are increased (Fig. 4a, c, d), reflecting the loss of RAS-MEK-MAPK-mediated cross-inhibition of PI3K-AKT-mTOR signaling [27]. Similarly, treatment with the PI3K inhibitor buparlisib led to a reduction in pAKT and pS6 levels, with loss of RAS-MEK-MAPK-mediated cross-inhibition, resulting in increased pERK levels (Fig. 4a–d). This concomitant upregulation of an alternative pathway explains why neither buparlisib nor trametinib alone inhibited tumor-cell proliferation (Fig. 4a, e). The trametinib–buparlisib combination readily inhibited both the RAS and PI3K pathways, leading to a significant, though modest, decrease in tumor-cell proliferation (Fig. 4). Thus, these two pathways appear to function redundantly in driving the proliferation of *nf1/pten*-mutant melanomas. Interestingly, 2 days of sirolimus treatment resulted in undetectable levels of pS6 staining (Fig. 4a, d), reflecting mTOR inhibition with transient increase and then sustained loss of pERK levels (Figs. 4a, b and 5) and suppression of proliferation (Fig. 4). Thus, the sustained compensatory upregulation of the ERK pathway induced by buparlisib was not evident when mTOR-mediated phosphorylation was specifically inhibited by sirolimus.

**Fig. 6:**
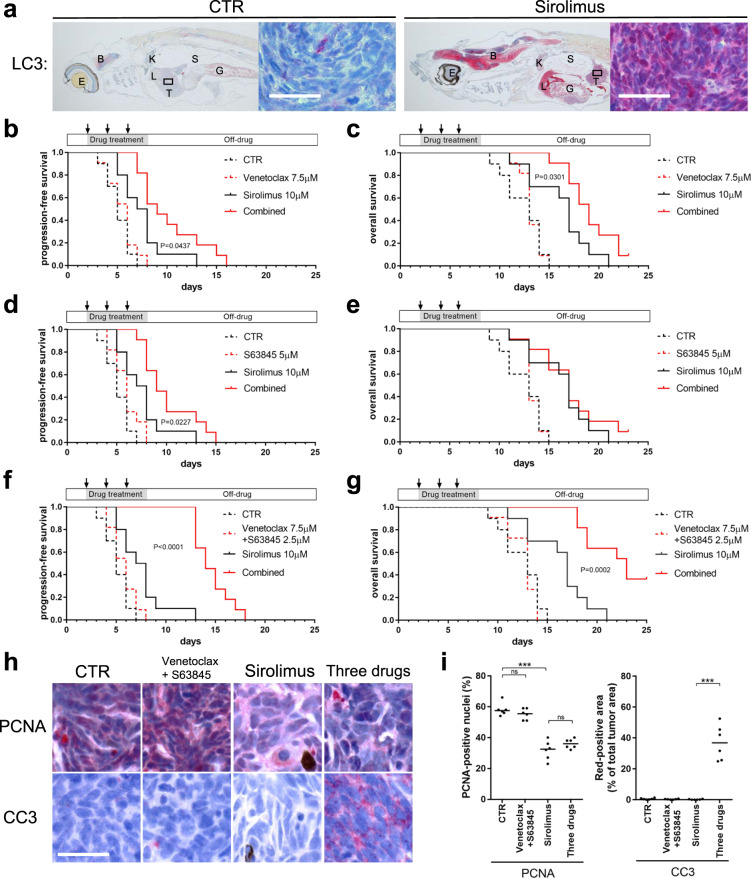
Sirolimus synergizes with venetoclax and S63845 to suppress *nf1/pten*-mutant melanoma tumor growth and extend the survival of tumor-bearing zebrafish. **a** Representative sagittal tissue sections from a transplanted *nf1/pten*-mutant melanoma tumor treated for 2 days with the indicated drugs. Sections were immunostained with antibodies to detect LC3A/B. *Left panels*: E = eye, B = brain, G = gut, K = kidney, L = liver, S = swim bladder, T = tumor. *Right panels*: ×63 magnification of tumor cells from the small black boxes in left panels. **b**–**g** Transplanted *nf1/pten*-mutant melanoma tumor cells were monitored daily in 3-week-old *rag2*^*−/−*^ recipient zebrafish treated with DMSO (CTR), venetoclax, S63845, sirolimus, or the drug combinations (*n* = 11 or 12 for each curve; doses as indicated). Kaplan–Meier curves for PFS (**b**, **d**, and **f**) and OS (**c**, **e**, and **g**) were compared using a log-rank test. Drugs were refreshed every 2 days during the 6-day course of treatment, as indicated by black arrows. **h** Representative tissue sections from transplanted *nf1/pten*-mutant melanoma tumors treated for 2 days with DMSO (CTR), 7.5 μM venetoclax and 2.5 μM S63845, 10 μM sirolimus, and the three-drug combination. Sections were immunostained using antibodies to detect PCNA and CC3 and quantified in (**i**). ns *p* > 0.05, ***p < 0.0001 by Mann–Whitney test. Scale bars = 20 μm.

To assess the durability of pathway suppression by inhibitor treatment, we treated *nf1/pten*-mutant-melanoma recipients with the inhibitors for 6 days, then analyzed the tumors after 4 days in the absence of the drugs. Sirolimus led to sustained reductions in pERK, pAKT, pS6, and PCNA levels at 4 days post treatment (Fig. 5a–c), as part of a cytoprotective autophagy stress response (Fig. 6a). By contrast, the initial signaling and antiproliferative effects of the trametinib-buparlisib combination (Fig. 4) were short-lived, as 4 days after drug removal, the pERK, pAKT, pS6, and PCNA levels were returning to normal (Fig. 5). Similar to sirolimus, temsirolimus also induced durable inhibition of pS6 and sustained suppression of pERK, pAKT, and tumor proliferation (Supplementary Fig. S13). Thus, in contrast to combined inhibition of PI3K and MEK, mTOR inhibition alone leads to the sustained suppression of RAS and PI3K pathways and tumor-cell growth in transplanted melanomas.

### Co-inhibition of BCL2 and MCL1 synergizes with sirolimus to cause apoptotic cell death *nf1/pten*-mutant melanomas in vivo

It is important to emphasize that while either sirolimus or temsirolimus can induce prolonged proliferative arrest based on the absence of PCNA staining, we did not detect cleaved caspase 3 in treated tumor cells (Figs. 4a, 5a, and S13), indicating that neither agent is cytotoxic as a single agent. To query further the proliferative arrest induced by these rapalogs, we studied the autophagy marker LC3 by IHC. This analysis revealed autophagy not only of the tumor cells by LC3 staining, but also striking levels of autophagy in the brain and liver of the sirolimus-treated animals (Fig. 6a). The absence of a cytotoxic effect and initiation of autophagy as a cell survival mechanism would likely limit the therapeutic potential of rapalogs in *nf1/pten*-mutant melanomas. Thus, we analyzed a panel of antitumor drugs to identify those with the potential to synergize with sirolimus by inducing apoptosis, thus converting “cytostatic autophagy” to “cytotoxic autophagy” [29]. This evaluation included MEK inhibitors trametinib and cobimetinib, the PI3K inhibitors buparlisib and apitolisib, the pan-RAF inhibitor sorafenib, the PARP inhibitor olaparib, the autophagy inhibitor chloroquine, and inhibitors of the BCL2 family of pro-survival proteins including sabutoclax, obatoclax, venetoclax, and S63845 (MTD determination see Supplementary Fig. S14).

As shown in Supplementary Fig. S15, none of the drugs delayed tumor progression when given alone to 3-week-old fish-bearing *nf1/pten*-mutant melanomas, and only sirolimus in combination with venetoclax showed overall survival benefit compared to sirolimus alone. In particular, the autophagy inhibitor chloroquine markedly delayed tumor progression when combined with sirolimus, presumably by blocking the ability of the autophagosomes to fuse with lysosomes, thus preventing both tumor and normal cells from accessing the nutrients sequestered in the autophagosome [30–32]. However, its use with sirolimus caused massive post-treatment death of the recipient fish as early as 4 days after drug administration, presumably due to autophagy of normal tissues such as liver (Figs. 6 and S15). Thus, we sought to identify drugs that would modify the autophagy response not directly as in the case of chloroquine but selectively by promoting apoptosis.

Pro-survival members of the BCL2 family of proteins are required for the survival of cells undergoing autophagy [33], with tumor cells typically showing greater dependence on these pro-survival effects because of their higher-than-normal expression of BH3-only initiators of apoptosis, leading to an increased propensity to undergo apoptosis through a mechanism called “apoptotic priming” [34]. Thus, since pro-survival BCL2 family proteins are essential in the high-stress environment induced by sirolimus, their inhibition would be expected to induce tumor cells to undergo apoptosis before normal cells [35–38]. Therefore, inhibitors of pro-survival BCL2 family proteins should have a therapeutic index based on synergy with the effects of sirolimus in targeted therapy for “primed” NF1/PTEN-mutant tumor cells, while sparing normal tissues.

To test this hypothesis, we focused on two inhibitors, venetoclax (inhibiting BCL2) [39] and S63845 (inhibiting MCL1) [40]. Interestingly, although venetoclax alone had no effect on tumor growth at a dose of 7.5 μM, its combination with 10 μM sirolimus significantly delayed tumor progression (Fig. 6b, c). Similarly, S63845 alone did not affect tumor growth at a dose of 5 μM, but in combination with 10 μM sirolimus, it augmented the growth suppressive effects of sirolimus (Fig. 6d, e).

It is known that each member of the pro-survival BCL2 family proteins, including BCL2 and MCL1, can bind and sequester BH3-only proteins independently and thereby prevent these BH3-only proteins from inducing apoptosis by activating BAX and BAK [39]. We previously discovered in vivo synergistic anti-leukemia activity of venetoclax and S63845, as each drug causes marked compensatory upregulation of MCL1 and BCL2 protein levels when used as single agent in zebrafish [41]. Hence, we reasoned that co-inhibition of BCL2 and MCL1 in *nf1/pten*-mutant melanoma cells might produce an even greater synergistic antitumor effect than observed with either inhibitor given individually with sirolimus. Indeed, when we combined 7.5 μM venetoclax and 2.5 μM S63845 with 10 μM sirolimus, we observed greatly enhanced growth suppression of *nf1/pten*-mutant melanoma cells (Fig. 6f, g). To determine the basis for this boosted effect, we analyzed the contributions of these three agents to tumor-cell proliferation and apoptosis. 7.5 μM venetoclax and 2.5 μM S63845 had no effect on proliferation or apoptosis, while 10 μM sirolimus significantly inhibited proliferation but failed to induce apoptosis (Fig. 6h, i). In combination, however, the three drugs effectively inhibited proliferation, and dramatically increased levels of apoptosis (Fig. 6h, i). Importantly, the fish tolerated this drug combination without noticeable toxicity. Thus, our results indicate that tumor cells sensitized by sirolimus become more dependent than normal cells on BCL2 and MCL1 for sustained survival, thus increasing their susceptibility to apoptosis in the absence of these key pro-survival proteins.

### Co-inhibition of BCL2 and MCL1 synergizes with sirolimus to induce apoptosis in human *NF1/PTEN*-deficient melanoma cells

**Fig. 7:**
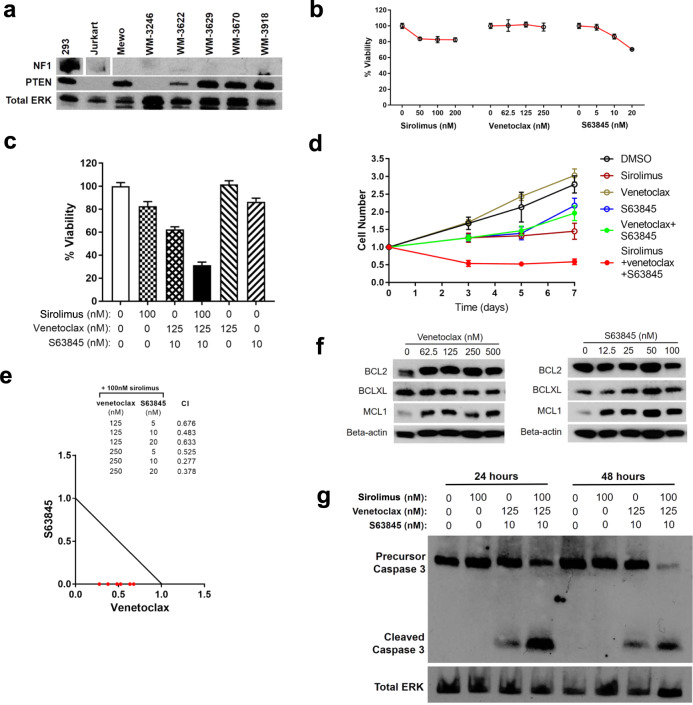
Venetoclax and S63845 synergize with sirolimus to induce apoptosis in human *NF1/PTEN*-deficient melanoma cells. **a** Western blots for NF1 and PTEN in a panel of human melanoma cell lines. HEK293 and Jurkart cells were included as positive and negative controls. The levels of total ERK1/2 expression serve as the loading control. **b** Relative cell viability of WM-3246 cells (Cell Titer Glo assay) upon treatment with sirolimus, venetoclax, or S63845 for 6 days. Mean ± s.d. values. **c** Relative cell viability of WM-3246 cells (Cell Titer Glo assay) upon treatment with the combination of sirolimus, venetoclax, and S63845 for 6 days. Mean ± s.d. values. **d** WM-3246 cell growth kinetics after treatment with the combination of sirolimus, venetoclax, and S63845 (for doses see panel c). Mean ± s.d. values. **e** Synergistic effects of venetoclax and S63845 on suppression of sirolimus-sensitized WM-3246 cells were analyzed by isobologram analysis. **f** Western blots for BCL2, BCLXL, and MCL1 in WM-3246 cells treated with venetoclax or S63845 for 24 h. **g** Western blots for cleaved caspase-3 in WM-3246 cells treated with the combination of sirolimus, venetoclax, and S63845.

To validate the efficacy of our three-drug combination, we turned to studies using human NF1/PTEN-deficient melanoma cells. For this purpose, we first evaluated the expression level of neurofibromin and PTEN in a panel of human melanoma cell lines and identified one cell line, WM-3246, that lacked detectable expression of either neurofibromin or PTEN (Fig. 7a). Then, using WM-3246 cells, we tested the effects of sirolimus, venetoclax and S63845 on the viability of NF1/PTEN-deficient melanoma cells. As a single agent, sirolimus induced only modest levels of cytostatic growth suppression at concentrations >50 nM (Fig. 7b–d). Venetoclax did not produce effects on WM-3246 cell growth at concentrations up to 250 nM, whereas S63845 suppressed cell growth in a dose-dependent manner at doses >5 nM (Fig. 7b, c). The greatest impact on cell growth was evident when sirolimus was tested in combination with venetoclax and S63845 (Fig. 7c, d); synergy was obtained by isobologram analysis over a range of drug concentrations (Fig. 7e), indicating that these cells depend on both BCL2 and MCL1, as well as on mTOR signaling, for cell growth and survival. Western blot analysis showed compensatory upregulation of MCL1 in cells treated with venetoclax (Fig. 7f), confirming the molecular basis for the synergy between S63845 and venetoclax in sirolimus-treated WM-3246 cells. Furthermore, cleaved caspase 3 in WM-3246 cells treated with the three-drug combination but not with sirolimus alone (Fig. 7g), validating the induction of apoptosis by co-inhibition of BCL2 and MCL1 in sirolimus-sensitized NF1/PTEN-deficient human melanoma cells.

We also tested the three-drug combination identified in our NF1/PTEN-mutant melanoma model in BRAF-mutant melanomas with PTEN mutations, because BRAF activation by mutation is more prevalent than biallelic inactivating mutations of NF1. Although each of these drugs demonstrated little or no activity as single agents, the three-drug combination showed significant activity against the BRAF-mutant melanoma cells harboring PTEN-mutation (Supplementary Fig. S16). Furthermore, the venetoclax-S63845 combination potentiated melanoma cell killing caused by the BRAFV600E inhibitor darafenib in BRAF-mutant melanoma cells (Supplementary Fig. S16), suggesting that co-inhibition of BCL2 and MCL1 as a strategy to enhance the induction of apoptosis has broad utility as a means to potentiate the activity of targeted therapies in disseminated human melanomas.

## Discussion

Loss-of-function mutations of the *NF1* tumor suppressor in human melanoma cells were first identified by us and others in the early 1990s [42, 43]. The TCGA program subsequently undertook a multiplatform characterization of cutaneous melanoma samples at the DNA, RNA, and protein levels, in which *NF1*-mutant melanoma emerged as an important subtype within a genomic classification framework [2]. Although highly useful as a means to identify cooperative molecular aberrations that might serve as druggable targets or predictive biomarkers, this genomic approach did not suggest a therapeutic strategy for tumors linked to *NF1* loss. Using a zebrafish experimental system that models human NF1-mutant melanomas, we show that activation of both the RAS and PI3K pathways in a background of pten loss is required to initiate melanomas in *nf1*-deficient animals. However, the RAS and PI3K pathways function redundantly in tumor maintenance, due to compensatory upregulation of either pathway when the other is inhibited (Figs. 4 and 5). Even simultaneous inhibition of both pathways only transiently inhibited the growth of *nf1/pten*-mutant melanomas, such that the overall survival of tumor-bearing fish was unaffected (Figs. 2, S7 and S10). This result contrasts with findings in basal-like breast cancer cell lines, in which the combination of MEK and PI3K inhibitors produced cytotoxic antitumor effects [44].

Given the superiority of sirolimus in suppressing the growth of transplanted *nf1/pten*-mutant melanomas while inducing autophagy in normal tissues, we faced a major challenge: to identify drugs that could selectively cause apoptosis in sirolimus-sensitized melanoma cells. Such studies require an animal model that allows one to simultaneously assess both antitumor effects and toxicity to normal tissues, a criterion that was readily met by our zebrafish model. Indeed, while the antitumor response of *nf1/pten*-mutant melanomas to the combination of sirolimus and chloroquine initially appeared promising, the treated fish died due to toxicity to normal tissues (Supplementary Fig. S15), illustrating the importance of analyzing this drug combination in an in vivo model system. By contrast, the combination of sirolimus with inhibitors of the anti-apoptotic proteins BCL2 (venetoclax) and MCL1 (S63845) was both well tolerated by normal tissues and highly active in inducing apoptosis in tumor cells (Figs. 6, 7, and S15). This selectivity apparently results from the fact that the malignant cells are “primed” to undergo apoptosis, while normal cells do not harbor the same levels of upregulation of BH3-only death proteins and can survive and maintain mitochondrial integrity despite the simultaneous inhibition of two major pro-survival proteins.

Our results underscore the advantages of using a reliable in vivo preclinical model to analyze the effects of simultaneously inhibiting multiple pathways with small-molecule drugs. Given its greater efficiency and lower costs compared to murine models, our zebrafish experimental system appears ideal for pursuing additional classes of pathway inhibitors in *NF1/PTEN*-mutant melanomas, as single agents and in combination, to define their clinical translational potential. Thus, the three-drug combination of sirolimus, venetoclax, and S63845 is well tolerated at effective dosages in vivo and shows activity against human as well as zebrafish NF1/PTEN-deficient melanoma cells, providing preclinical evidence justifying an early-stage clinical trial in patients with melanomas of this high-risk genomic subtype. Notably, the three-drug combination identified in our NF1/PTEN-mutant melanoma model also showed anti-melanoma activity in BRAF-mutant melanoma cells harboring PTEN-mutation (Supplementary Fig. S16). Furthermore, the venetoclax-S63845 combination potentiated melanoma cell killing caused by the BRAFV600E inhibitor darafenib in BRAF-mutant melanoma cells (Supplementary Fig. S16). Thus the potentiation of apoptosis induced by co-inhibition of BCL2 and MCL1 is a strategy with wide applicability to enhance the anti-melanoma activity by targeted therapies in malignant melanoma.

## Materials and methods

### Zebrafish

Zebrafish experiments and animal husbandry were performed in accordance with Dana-Farber Cancer Institute IACUC-approved protocol #02-107.

### Melanoma tumor watch

*nf1a*^*+/−*^*;nf1b*^*+/−*^*;ptena*^*+/−*^*;ptenb*^*+/−*^*;p53*^*+/M214K*^ mutant zebrafish were incrossed, and offspring were monitored every week, starting at 3 weeks, for hyperpigmented cell masses indicative of melanoma tumors. Once a hyperpigmented cell mass was identified, the individual fish was separated and carefully monitored weekly for at least 3 weeks for tumor progression. Only fish with expanding hyperpigmented cell masses were scored as tumor fish and analyzed further by H&E staining and immunohistochemical assays. All fish were genotyped for *nf1a*, *nf1b*, *ptena*, *ptenb*, and *p53* at the age of 6 weeks. The exact sample size (*n*) for each experimental group is indicated in the figures.

### Tumor-cell transplantation

*rag2*^*E450fs*^*(casper)* (*rag2*^*−/−*^*)* zebrafish were anaesthetized with 0.003% tricaine (Sigma-Aldrich, St. Louis, MO) and positioned on a 10-cm Petri dish coated with 1% agarose. Primary and serially passaged tumors derived from *nf1a*^*+/−*^*;nf1b*^*−/−*^*;ptena*^*+/−*^*;ptenb*^*−/−*^*;p53*^*M214K/M214K*^ and *nf1a*^*+/−*^*;nf1b*^*−/−*^*;ptena*^*+/−*^*;ptenb*^*−/−*^*;p53*^*M214K/M214K*^*;Tg(sox10:EGFP)* zebrafish lines were excised from tumor-bearing fish and mechanically dissociated with a razor blade in 0.9× PBS + 5% FBS (Life Technologies, Carlsbad, CA) at room temperature. The collected cell suspension was filtered through a 40-μm cell strainer (Falcon, Corning, NY) and resuspended in 0.9× PBS + 5% FBS. For the intraperitoneal and intramuscular transplantation into 3- to 4-month-old adult *rag2*^*−/−*^ fish, a 26 s/2″/2 Hamilton 80300 syringe (Hamilton, Reno, NV) was used [23]. For the intraperitoneal transplantation into 3-week-old juvenile *rag2*^*−/−*^ fish, cell suspensions were loaded into borosilicate glass capillary needles (1 mm o.d. × 0.78 mm i.d.; Harvard Apparatus, Holliston, MA), and the injections were performed with a Pneumatic Picopump and a manipulator (WPI, Sarasota, FL) [45].

### Cell culture

Melanoma cell lines Mewo, WM-3246, WM-3622, WM-3629, WM-3670 and WM-3918 were purchased from Rockland (Rockland Immunochemicals Inc, Limerick, PA), and maintained in Dulbecco’s modified Eagle’s medium supplemented with 10% FBS, l-glutamine, and penicillin/streptomycin. Melanoma cell lines COLO829 and C32 were purchased from ATCC (ATCC, Manassas, VA) and maintained according to the provided Culture Methods. HEK-293T cells were purchased from ATCC, and maintained in Dulbecco’s modified Eagle’s medium supplemented with 10% FBS, l-glutamine, and penicillin/streptomycin. Jurkart cells were maintained in RPMI-1640 medium supplemented with 10% FBS, l-glutamine, and penicillin/streptomycin. The identity of cell lines used in this study was verified by short tandem repeat analysis using the PowerPlex 1.2 system (Promega). The cell lines were tested for mycoplasma contamination using MycoAlert Mycoplasma Detection Kits (Lonza).

### Statistical analysis

Statistical analysis was performed with Prism 5 software (GraphPad). Kaplan-Meier methods and the log-rank test were applied to assess the rate of tumor growth in Figs. 1 and S1, and tumor progression in Figs. 2, 6, S7, S8, S9, S10 and S15. The quantitative data in Figs. 3, 4, 5 and S12 are reported as median values. A Mann–Whitney test with confidence intervals of 95% was used for the analyses in Figs. 3, 4, 5, 6 and S12.

## Supplementary information

Supplementary figures

supplementary material
