# Supplementary material for: Synergistic melanoma cell death mediated by inhibition of both MCL1 and BCL2 in high-risk tumors driven by NF1/PTEN loss
Source: Oncogene. 2021 Jul 30;40(38):5718–29. doi: 10.1038/s41388-021-01926-y (PMC8460449; doi:10.1038/s41388-021-01926-y)
Supplement: Supplementary file 1 — Supplementary figures [file 41388_2021_1926_MOESM1_ESM.pptx]

## Slide 1
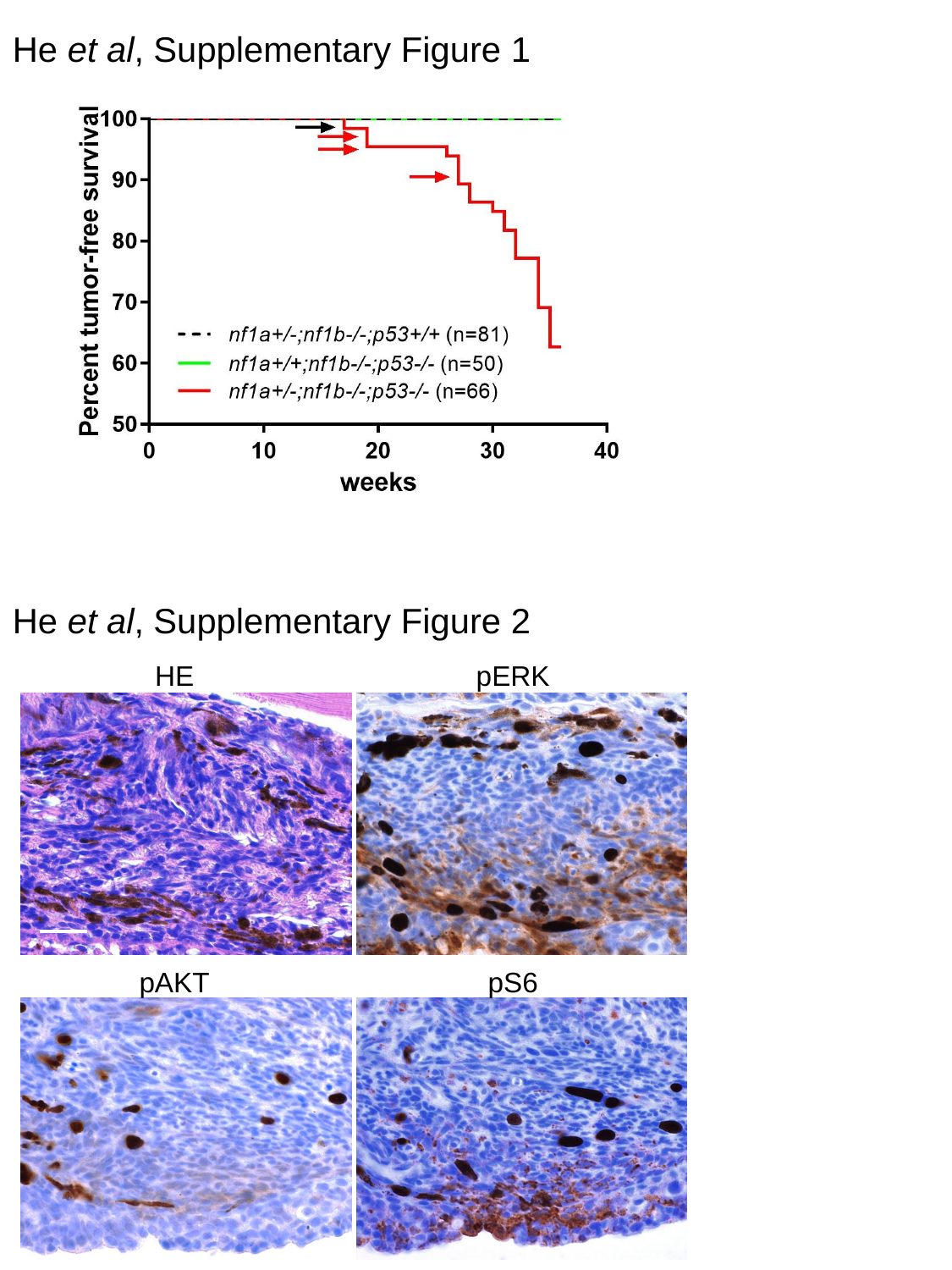

He et al, Supplementary Figure 1
He et al, Supplementary Figure 2
HE
pERK
pAKT
pS6

## Slide 2
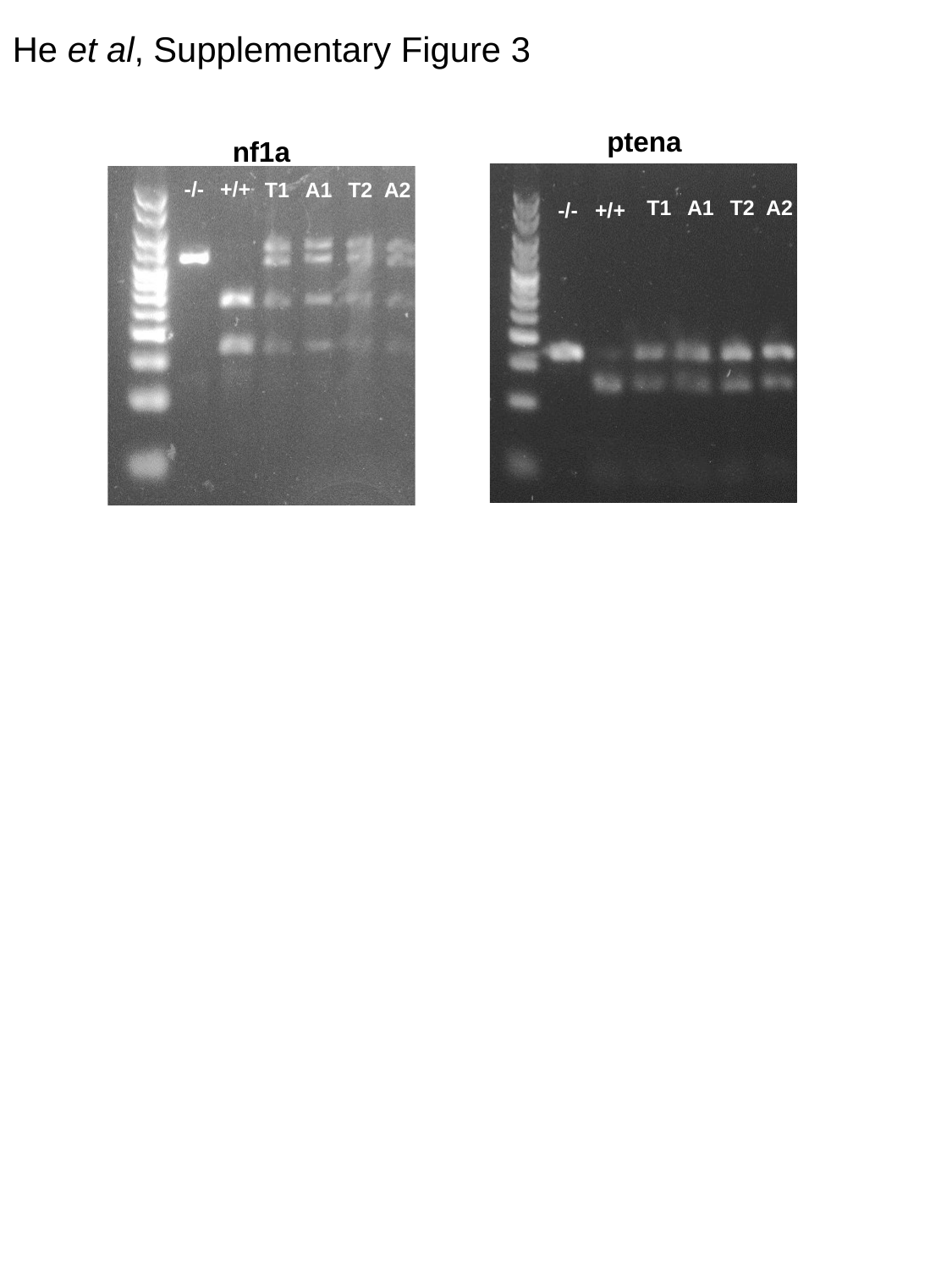

He et al, Supplementary Figure 3
ptena
nf1a
-/-
+/+
T1
A1
T2
A2
T1
A1
T2
A2
-/-
+/+

## Slide 3
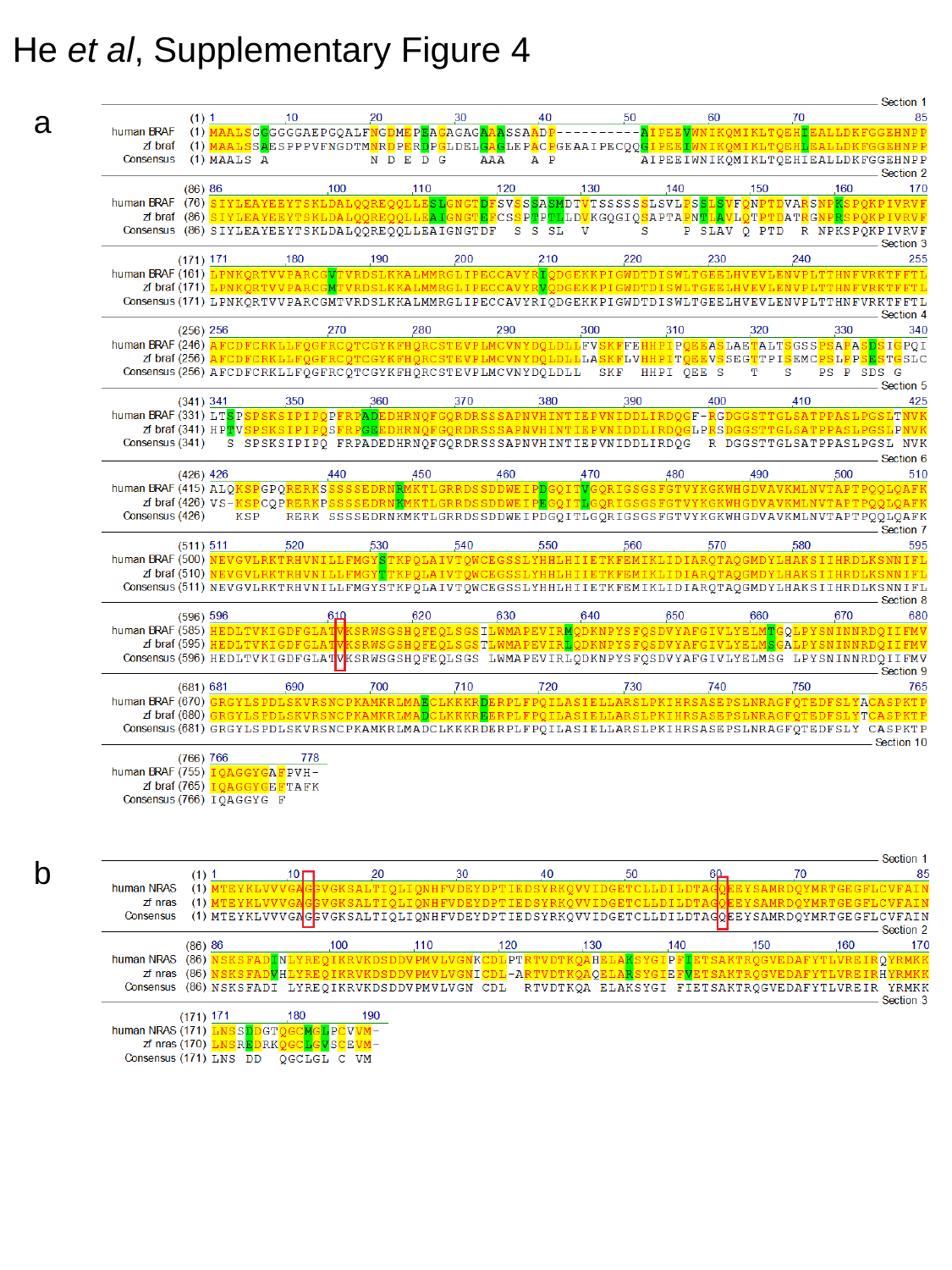

He et al, Supplementary Figure 4
a
b

## Slide 4
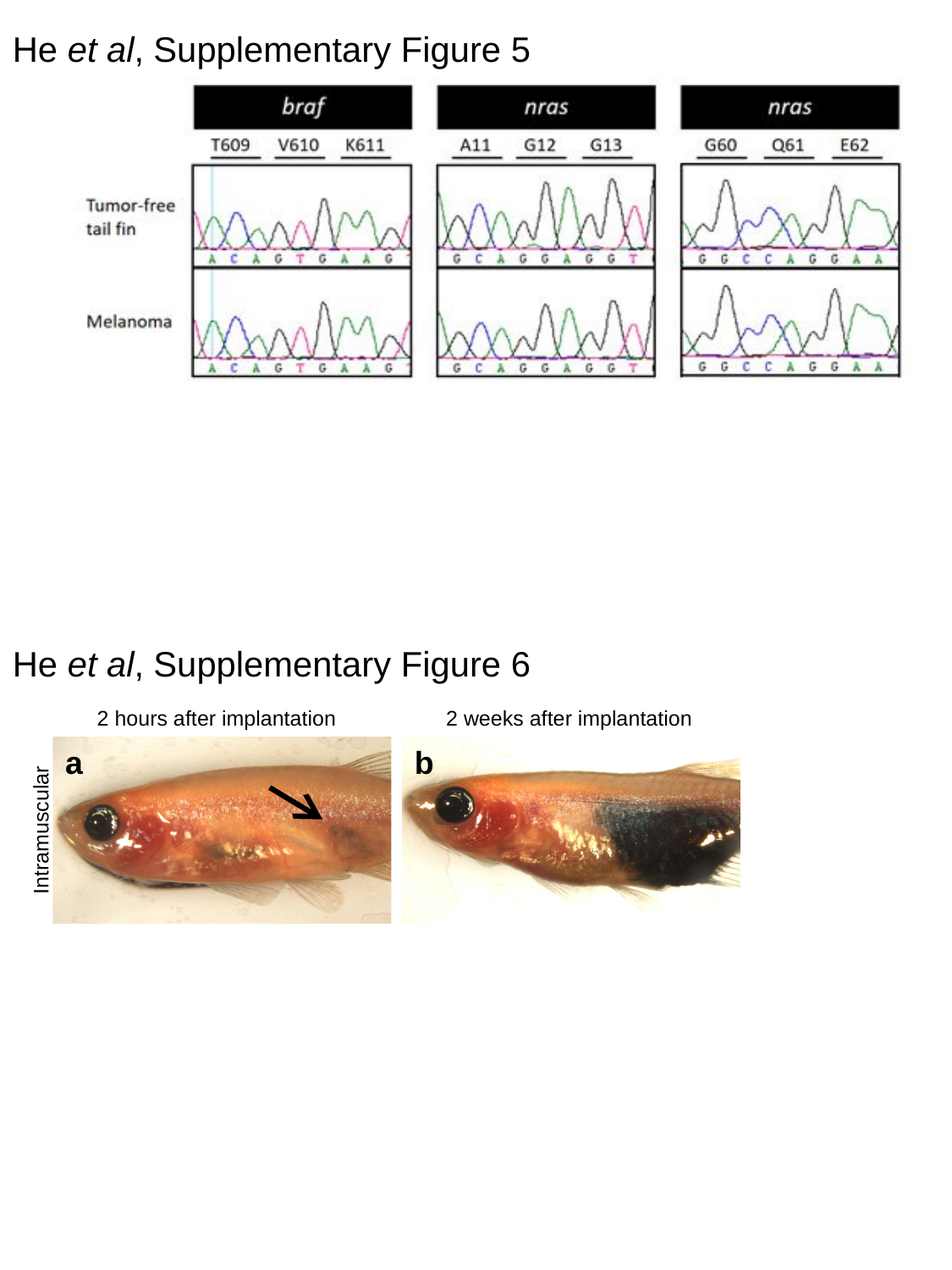

He et al, Supplementary Figure 5
He et al, Supplementary Figure 6
2 hours after implantation
2 weeks after implantation
a
b
Intramuscular

## Slide 5
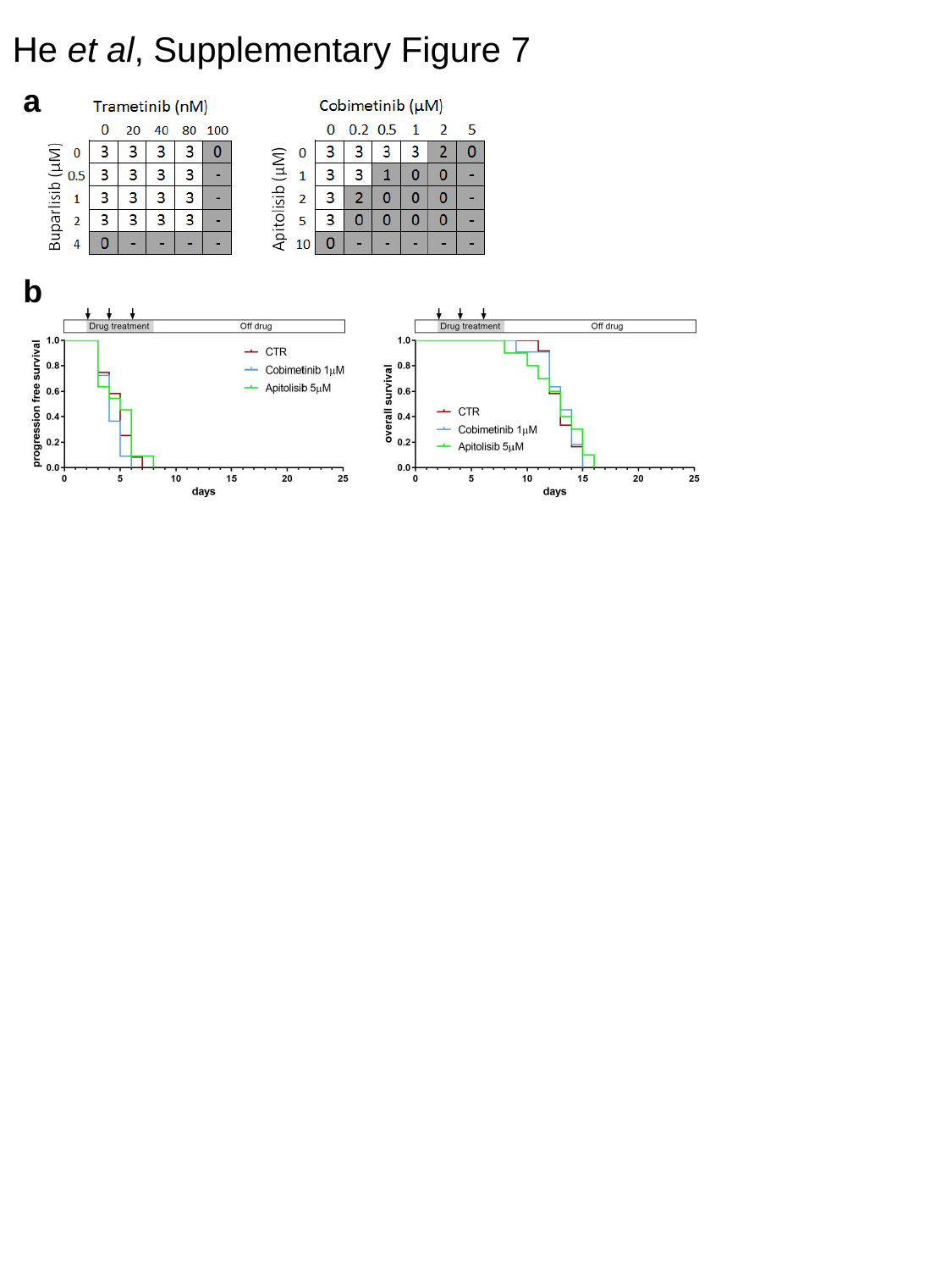

He et al, Supplementary Figure 7
a
b

## Slide 6
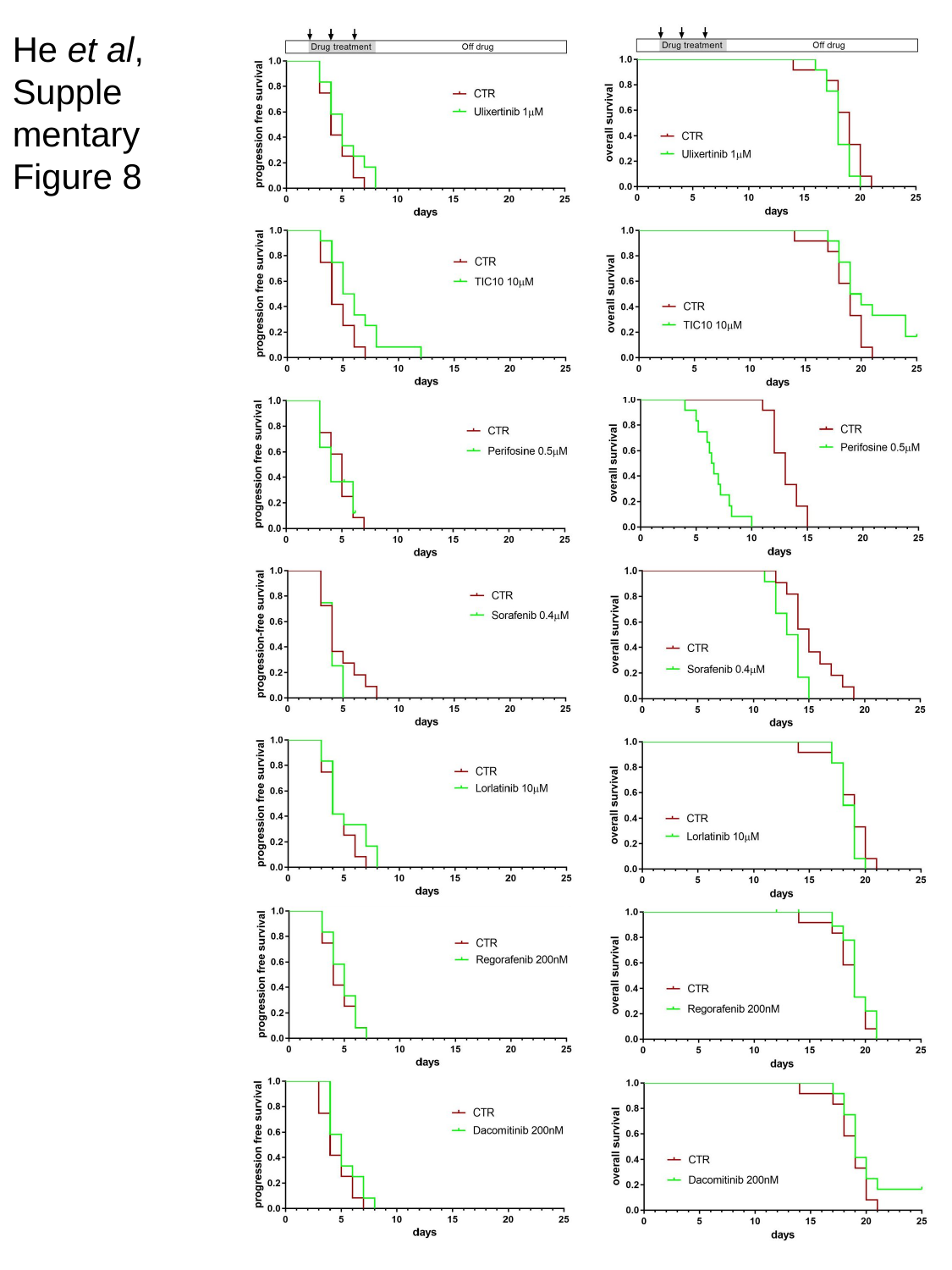

He et al, Supplementary Figure 8

## Slide 7
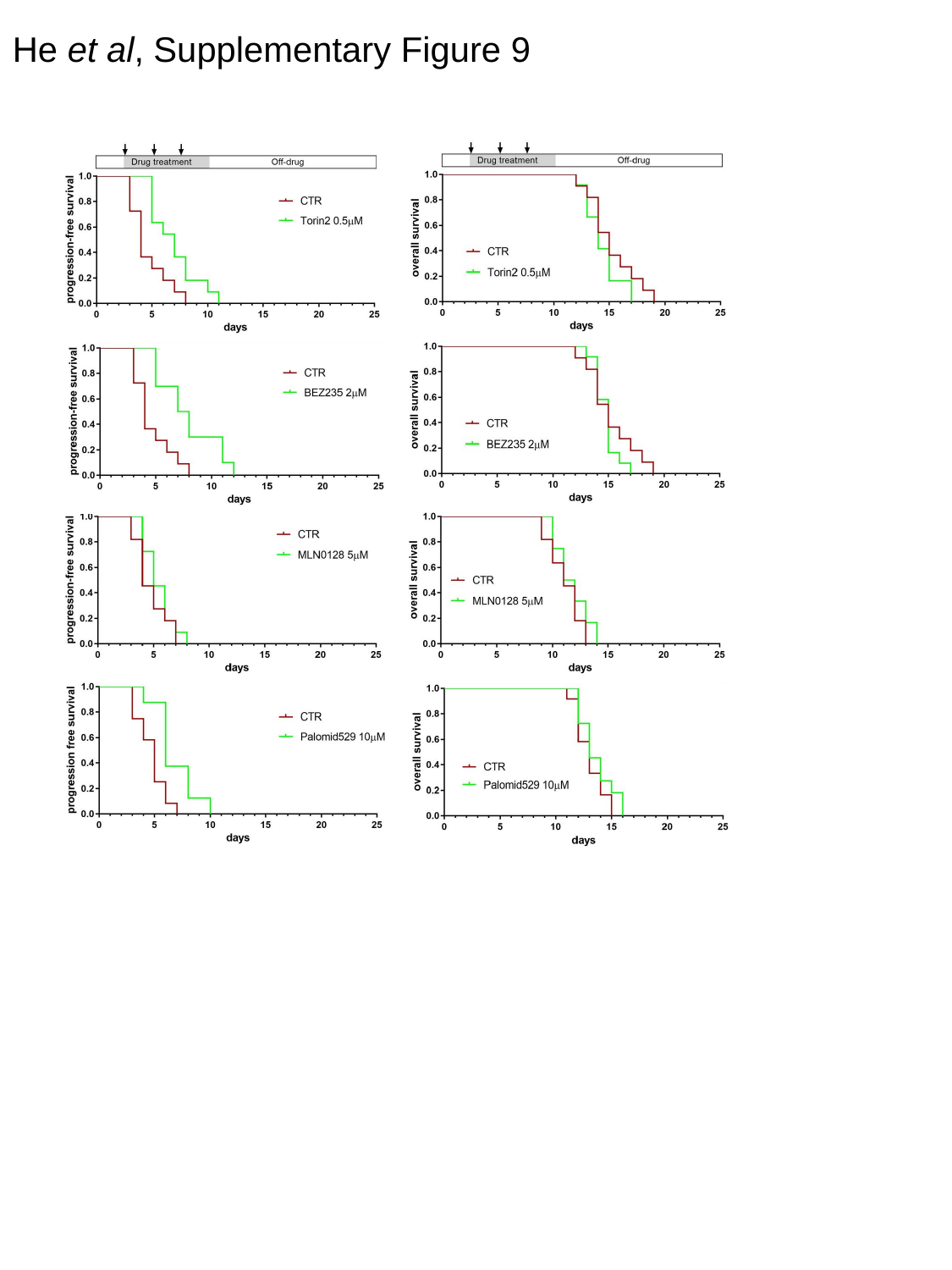

He et al, Supplementary Figure 9

## Slide 8
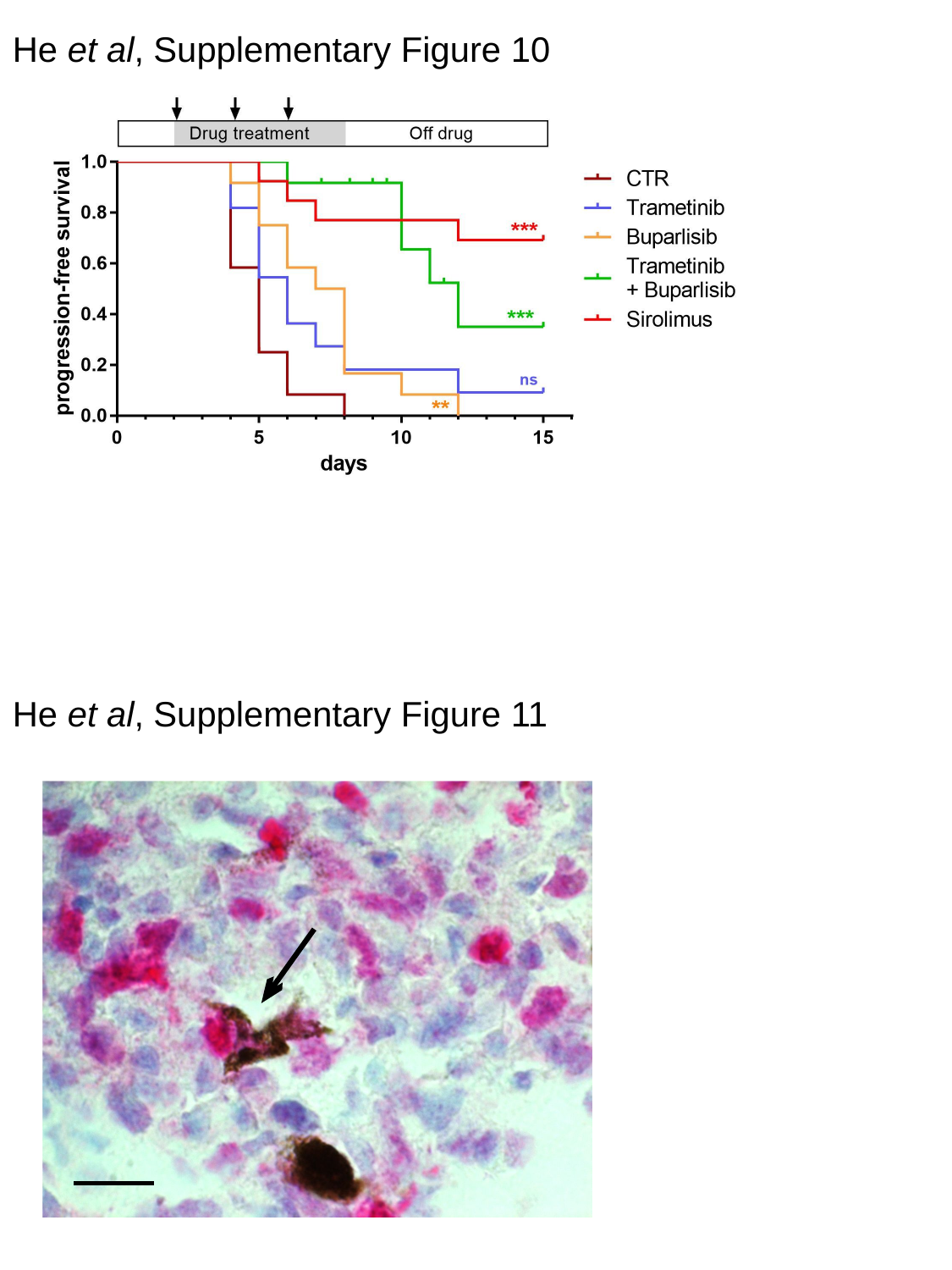

He et al, Supplementary Figure 10
He et al, Supplementary Figure 11

## Slide 9
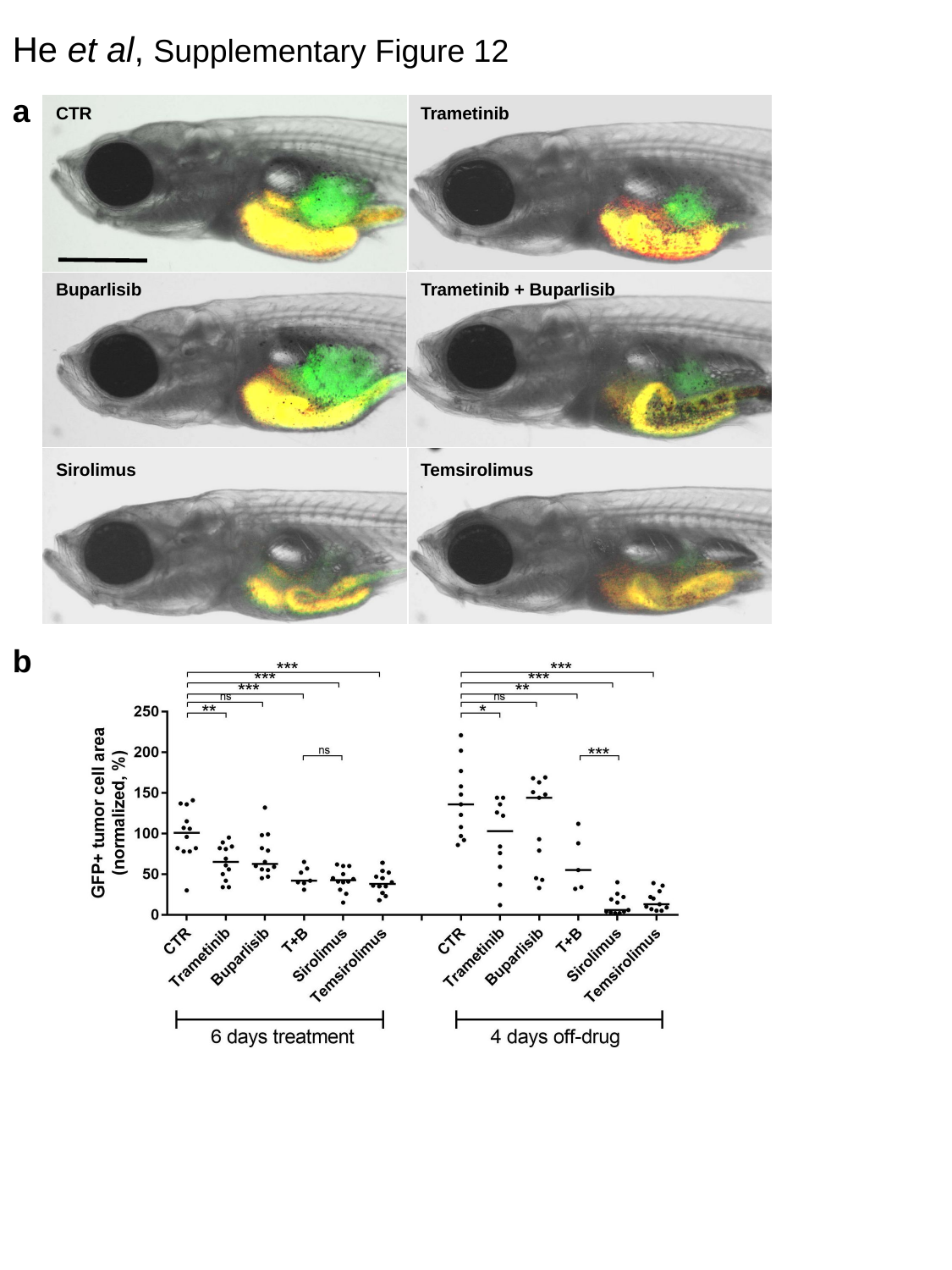

He et al, Supplementary Figure 12
a
CTR
Trametinib
Buparlisib
Trametinib + Buparlisib
Sirolimus
Temsirolimus
b

## Slide 10
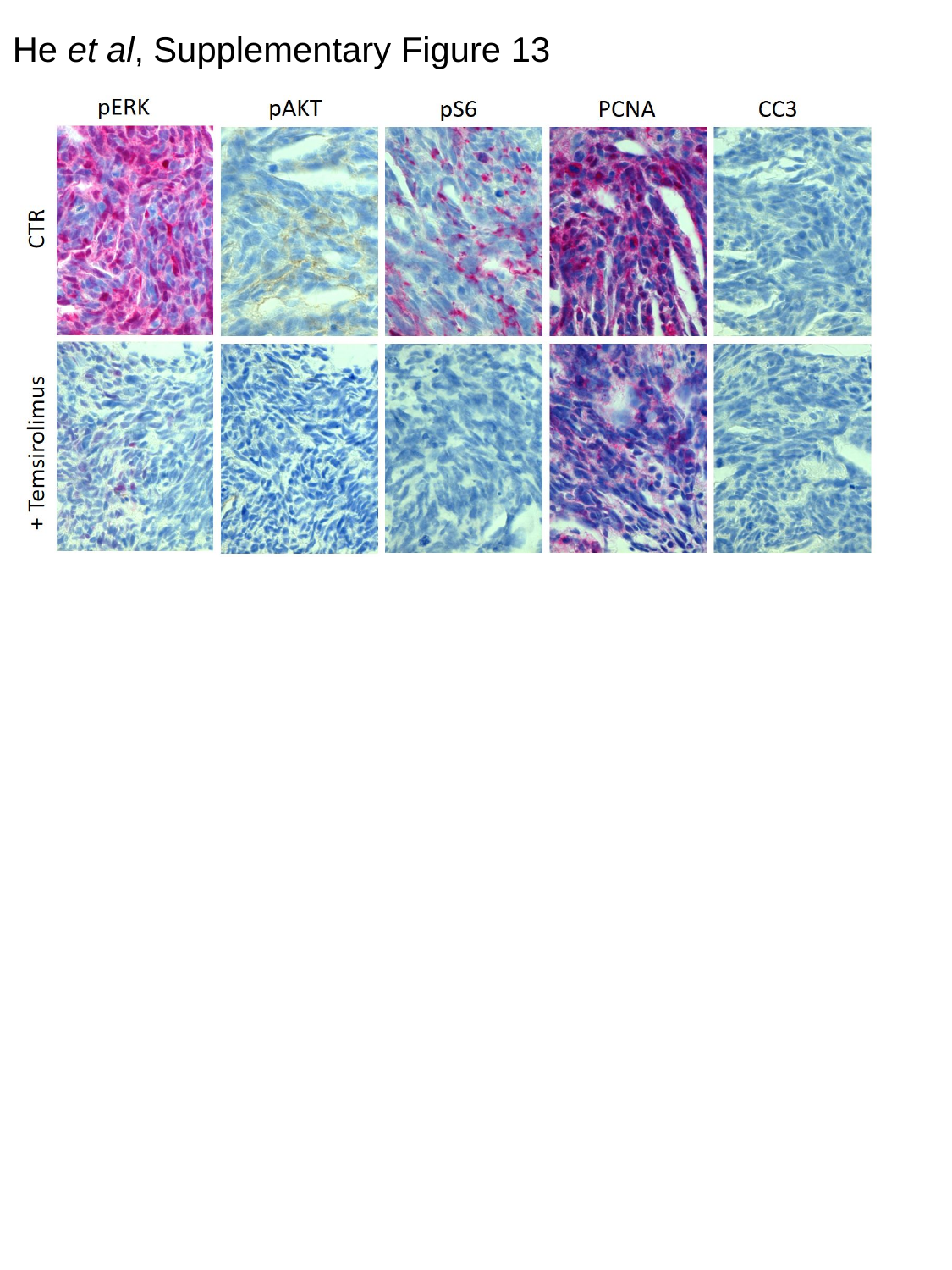

He et al, Supplementary Figure 13

## Slide 11
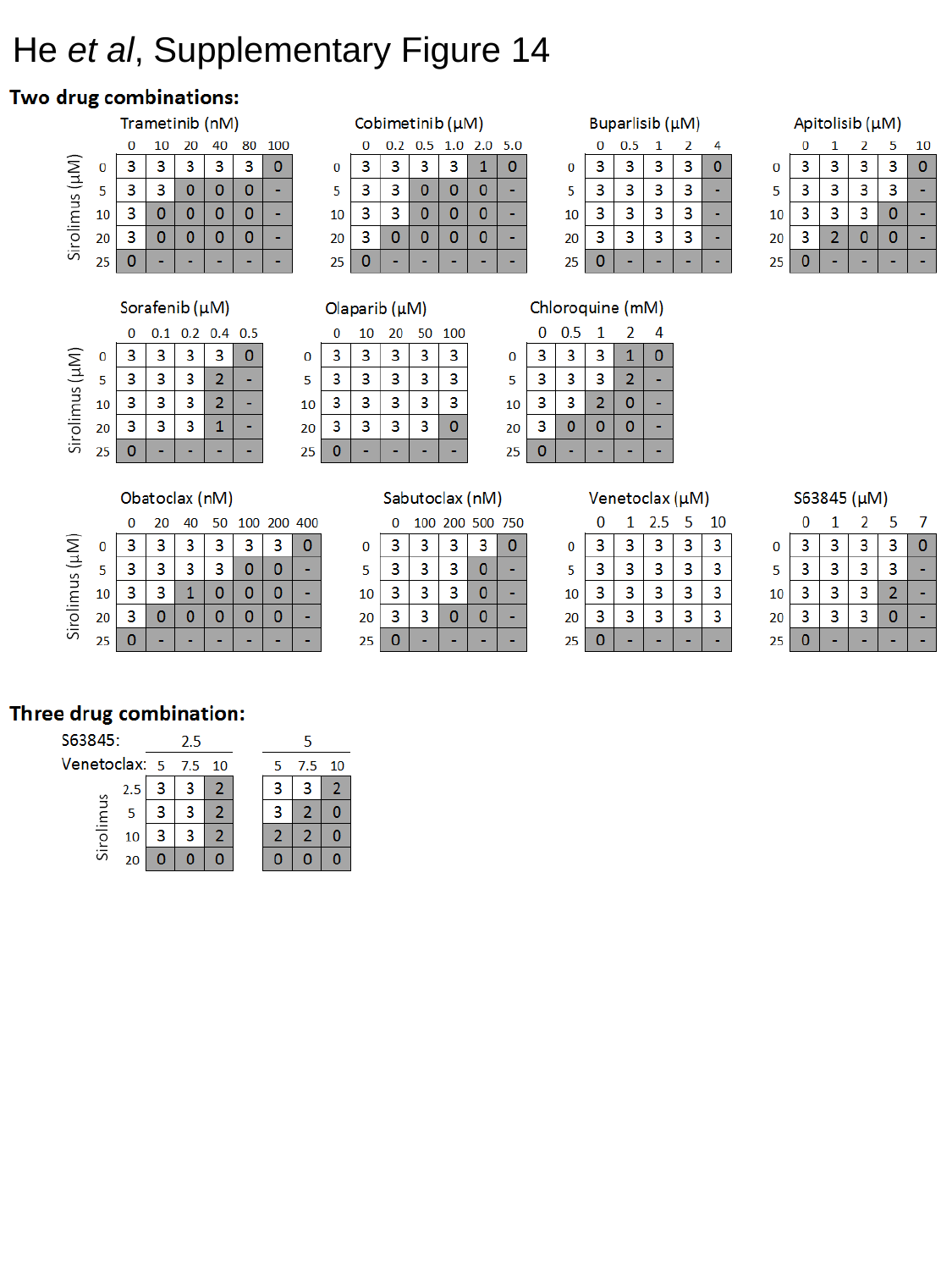

He et al, Supplementary Figure 14

## Slide 12
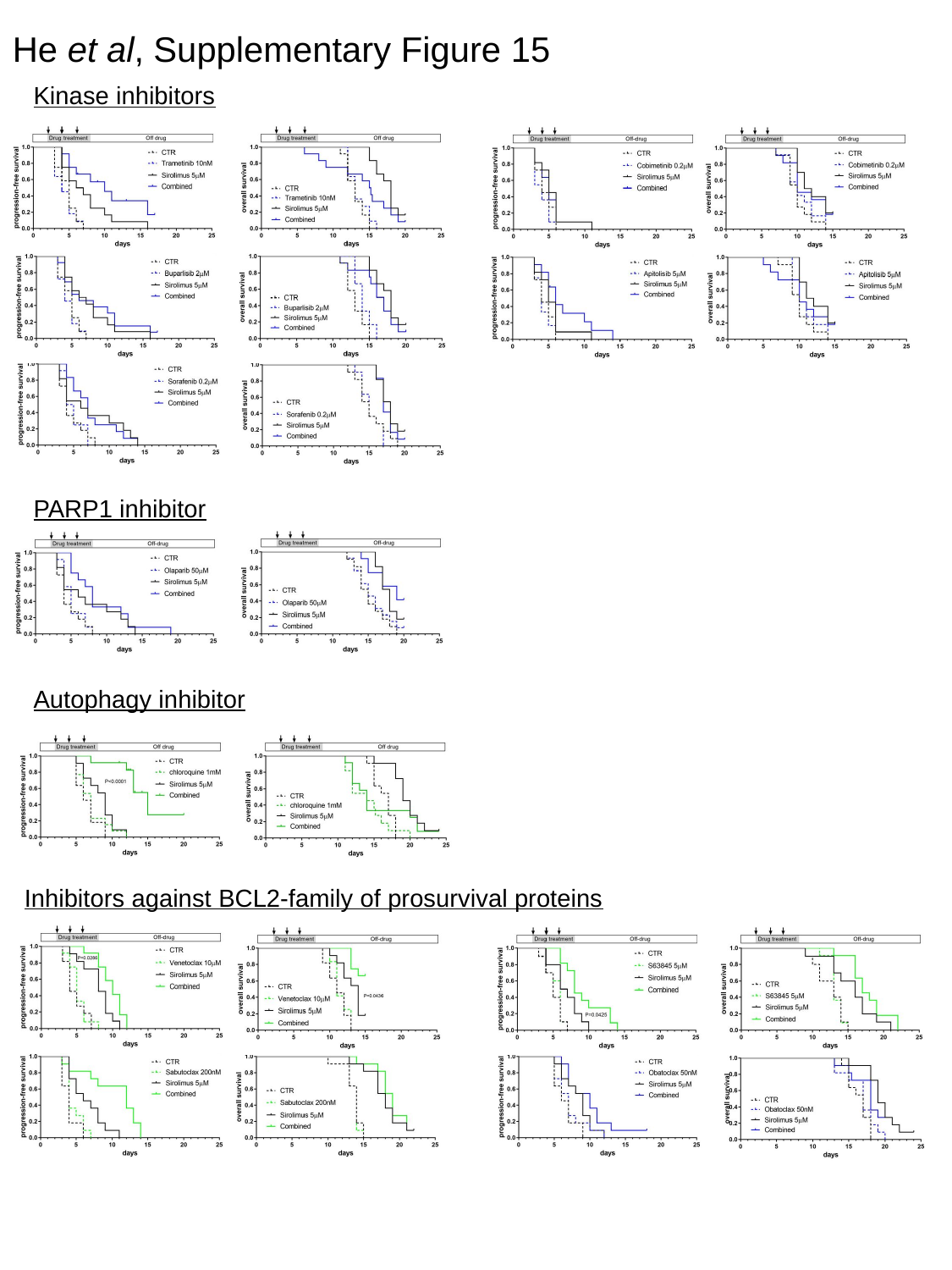

He et al, Supplementary Figure 15
Kinase inhibitors
PARP1 inhibitor
Autophagy inhibitor
Inhibitors against BCL2-family of prosurvival proteins

## Slide 13
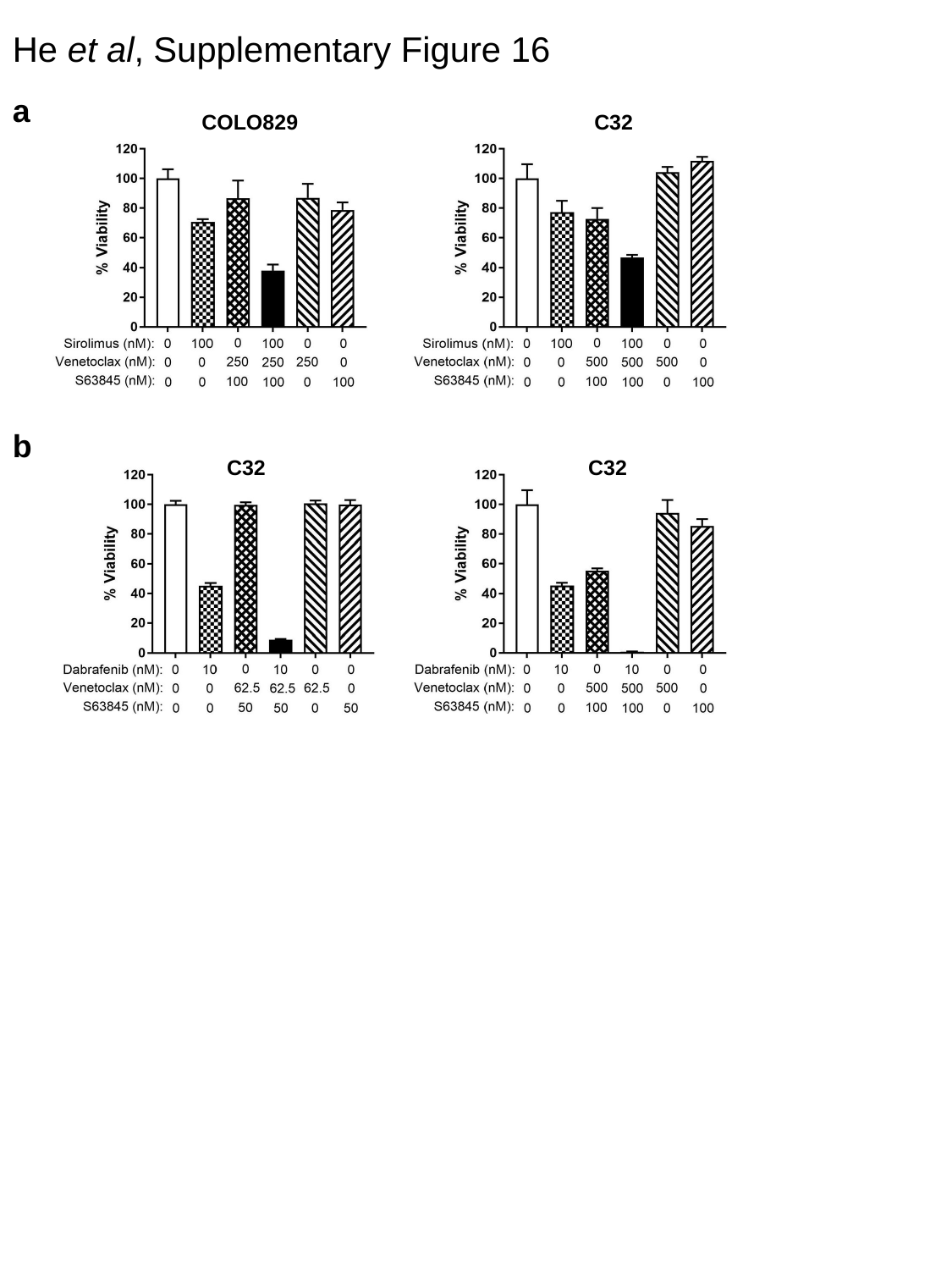

He et al, Supplementary Figure 16
a
C32
COLO829
b
C32
C32
