# Supplementary material for: Synergistic melanoma cell death mediated by inhibition of both MCL1 and BCL2 in high-risk tumors driven by NF1/PTEN loss
Source: Oncogene. 2021 Jul 30;40(38):5718–29. doi: 10.1038/s41388-021-01926-y (PMC8460449; doi:10.1038/s41388-021-01926-y)
Supplement: Supplementary file 2 — supplementary material [file 41388_2021_1926_MOESM2_ESM.docx]

Supplementary Materials

**Supplementary Figure 1**: Cumulative frequency of tumor development in fish with the indicated genotypes. Malignant peripheral nerve sheath tumors (MPNSTs) comprise the majority of tumors; high grade gliomas are indicated by red arrows with the single black arrow indicating a spontaneous melanoma.

**Supplementary Figure 2**: Sections from a spontaneous melanoma from a 20-week-old *nf1a^+/-^;nf1b^-/-^;ptena^+/-^;ptenb^-/-^;p53^+/M214K^* zebrafish were stained for H&E, pERK, pAKT, pS6, and PCNA (63x magnification, Scale bar = 20 μm).

**Supplementary Figure 3**: DNA PCR of *nf1a* and *ptena* genes were performed from two pairs of independent melanoma tumors (T1 and T2) and the corresponding adjacent non-tumor muscle tissue (A1 and A2). The PCR products were then cut with restriction enzymes that only digests the wild-type allele but not the mutant allele (DdeI for *nf1a* and RsaI for *ptena*). Electrophoresis was performed using 3% MetaPhor agarose. The results showed that the wild-type allele of each gene is retained by the tumor cells.

**Supplementary Figure 4**: Amino acid sequence alignment of human and zebrafish BRAF (a) and NRAS (b). The sites of BRAF codons V600, and NRAS codons G12 and Q61, are highlighted by red boxes.

**Supplementary Figure 5**: Representative sequencing chromatograms of zebrafish codons, shown are *braf* codon V610, and *nras* codons G12 and Q61 from genomic DNA isolated from a melanoma tumor (bottom) and matched tumor-free tail fin (top).

**Supplementary Figure 6**: Rapid growth of *nf1/pten*-mutant melanoma following intramuscular engraftment into adult *rag2^-/-^* Casper zebrafish. The implanted melanoma cells (a, indicated by the arrow) grew aggressively into secondary tumors within 2 weeks (b).

**Supplementary Figure 7**: (a) Dose matrices were generated to assess the tolerability of 3-week-old zebrafish to MEK and PI3K inhibitors. Each matrix sampled mixtures of 2 serially diluted single-agent concentrations. Three 3-week-old wild-type zebrafish were independently treated with each mixture for 7 days, with drug refreshments at days 2 and 4. The numbers of fish surviving the treatment was measured at the end of day 7. (b) Transplanted *nf1/pten*-mutant melanoma tumor cells were monitored daily in 3-week-old *rag2^-/-^* recipient zebrafish treated with DMSO (CTR; n=12), 1 μM cobimetinib (n=11) and 5 μM apitolisib (n=11) for 20 days. Kaplan-Meier curves for PFS (left) and OS (right) were compared using a log-rank test.

**Supplementary Figure 8**: Transplanted *nf1/pten*-mutant melanoma cells were monitored daily in 3-week-old *rag2^-/-^* recipient zebrafish treated with DMSO (CTR) or selected drugs (n=11 or 12 for each curve). Kaplan-Meier curves for progression-free (left) or overall (right) survival were compared with a log-rank test.

**Supplementary Figure 9**: Transplanted *nf1/pten*-mutant melanoma cells were monitored daily in 3-week-old *rag2^-/-^* recipient zebrafish treated with DMSO (CTR) or selected mTOR inhibitors (n=11 or 12 for each curve). Kaplan-Meier curves for progression-free (left) or overall (right) survival were compared with a log-rank test.

**Supplementary Figure 10**: Transplanted *nf1/pten*-mutant melanoma tumor cells were monitored daily in 3-week-old *rag2^-/-^* recipient zebrafish treated with DMSO (CTR; n=12), 80 nM trametinib (n=11), 2 μM buparlisib (n=12), the combination of 80 nM trametinib and 2 μM buparlisib (n=11), or 20 μM sirolimus (n=12) for 15 days. Kaplan-Meier curves for progression-free (left) or overall (right) survival were compared with a log-rank test (ns p>0.05, *p<0.05, **p<0.01, ***p<0.001).

**Supplementary Figure 11**: Representative tissue section from a transplanted melanoma tumor derived from *nf1a^+/-^;nf1b^-/-^;ptena^+/-^;ptenb^-/-^;p53^M214K/M214K^;Tg(sox10:EGFP)* zebrafish immunostained with an antibody to GFP. Within the amelanotic tumor mass, a GFP-expressing, melanin-positive melanoma cell is indicated by the arrow. Scale bar = 20μm.

**Supplementary Figure 12**: **mTOR inhibitors sirolimus and temsirolimus produce a durable antitumor effect on amelanotic *nf1/pten*-mutant melanoma cells.** (a) EGFP-positive amelanotic melanoma cells were isolated from *nf1a^+/-^;nf1b^-/-^;ptena^+/-^;ptenb^-/-^;p53^M214K/M214K^;sox10:EGFP* zebrafish and injected intraperitoneally into 3-week-old *rag2^-/-^* zebrafish. Starting at 2 days post-transplantation, the recipient fish were treated with DMSO (CTR), 80 nM trametinib, 2 μM buparlisib, the combination of 80 nM trametinib and 2 μM buparlisib, 20 μM sirolimus, or 40 μM temsirolimus for 6 days. EGFP-expressing tumors were photographed at 4 days post-treatment. The EGFP-expressing area appears as green while the autofluorescence in the gastrointestinal tract appears as yellow. Scale bar = 1 mm. (b) Quantification of the area of EGFP tumor fluorescence immediately after the 6-day course of drug treatment (left) and 4 days later (right). “T+B” refers to trametinib plus buparlisib. ns p>0.05, *p<0.05, **p<0.01, ***p<0.001 by two-tailed unpaired t-test.

**Supplementary Figure 13**: Representative tissue sections from a transplanted *nf1/pten*-mutant melanoma at 4 days after a 6-day drug treatment with DMSO (CTR) and 40 μM temsirolimus. Sections were immunostained with antibodies to detect pERK, pAKT, pS6, PCNA and CC3.

**Supplementary Figure 14**: Dose matrices were generated to assess the tolerability of 3-week-old zebrafish to sirolimus combined with the indicated drugs. Each matrix sampled mixtures of 2 serially diluted single-agent concentrations. Three 3-week old wild-type zebrafish were independently treated with each mixture for 7 days, with drug replenishment at days 2 and 4. The numbers of fish surviving the treatment was measured at the end of day 7.

**Supplementary Figure 15**: Three-week-old *rag2^-/-^* recipient zebrafish were transplanted with *nf1/pten*-mutant melanoma cells and treated with selected drugs as single agents or in combination with 5 μM sirolimus (n=11 or 12 for each curve). Kaplan-Meier curves for progression-free (left) or overall (right) survival were compared with a log-rank test. Drugs were replenished every 2 days during the 6-day course of treatment (black arrows).

**Supplementary Figure 16**: (a) Venetoclax and S63845 synergize with sirolimus to suppress human melanoma cells harboring BRAFV600E and PTEN mutations. Relative cell viability of COLO829 and C32 cells (Cell Titer Glo assay) upon treatment with the combination of sirolimus, venetoclax and S63845 for 3 days. Mean ± s.d. values. (b) Venetoclax and S63845 synergize with dabrafenib to kill BRAF-mutant melanoma cells. Relative cell viability of C32 cells (Cell Titer Glo assay) upon treatment with the combination of dabrafenib, venetoclax and S63845 for 3 days. Mean ± s.d. values.

**Supplementary Material and Methods**

**DNA extraction, PCR and sequencing**

From each *nf1a^+/-^;nf1b^-/-^;ptena^+/-^;ptenb^-/-^;p53 ^M214K/M214K^* fish, the melanoma tumor and the tumor-free tail fin were excised, and genomic DNA was extracted with 30 μL QuickExtract DNA Extraction Solution (Epicentre, Madison, WI) according to the manufacturer’s instructions. PCR reactions were performed in a 25 μL volume consisting of 4 μL genomic DNA, 1.4 μL 2.5 mM dNTP (Invitrogen, Carlsbad, CA), 0.16 μL SuperTaq enzyme (NEB, New England Biolabs, Ipswich, MA), 2.5 μL 10x SuperTaq buffer (NEB) and 1.4 μL 10 μM primer mix. Cycling parameters were 1) 94°C for 2 min, 2) 40 cycles at 94°C for 30 sec, annealing (temperature depends on primer sets, see below) for 30 sec, and elongation at 72°C for 30 sec, and 3) final elongation for 5 min at 72°C. PCR primer sequences and annealing temperatures were as follow: *nf1a* forward, 5’-GGTGTGTATGTAAATGGGCTCAATG-3’ and reverse 5’-TACAGTTTCCATAAAACCTGACATTTC-3’ (62°C); *ptena* forward, 5’- TTGCCATGGGCTTTCCAGCCGTA-3’ and reverse 5’-CCACGTTGACTTACCGGACAACGTCA-3’ (53°C); *brafV610* forward, 5’-ATTAGCCGTAACATCACTTCTCTAG-3’ and reverse 5’-ATGTAAGATGTGTTCCTTCACTCAC-3’ (53°C); *nrasG12* forward, 5’- gcttactctctgtctttaattac-3’ and reverse 5’- aagtatagtaaatttcctcat-3’ (53°C); *nrasQ61* forward, 5’-gtggcaatcttgtctttc-3’ and reverse 5’-ctgctctcagacCTGTAC-3’ (60°C). Sequencing of the PCR reaction products were performed by Genewiz (Cambridge, MA).

**Immunohistochemistry**

Zebrafish were euthanized in tricaine anesthetic, fixed in 4% paraformaldehyde at 4°C for 2 days, and decalcified with 0.25 M EDTA, pH 8.0, for at least 24 hr. Paraffin sectioning followed by H&E staining or IHC was performed at the Dana-Farber/Harvard Cancer Center Research Pathology Core. Primary antibodies included phospho-p44/42 MAPK (ERK1/2) (Thr202/Tyr204, Cell Signaling #4370; 1:150), phospho-AKT (Ser473, Cell Signaling #4060), phospho-S6 ribosomal protein (Ser240/244, Cell Signaling #4838), PCNA (PC10, EMD Millipore; 1:100), cleaved caspase-3 (Cell Signaling #9664; 1:100), GFP (Abcam #6556, 1:150), LC3A/B (Cell Signaling #12741; 1:200), HSP90 (Cell Signaling #4874; 1:100), and HSP70 (Enzo #ADI-SPA-810; 1:25). Antibody binding was detected with either a diaminobenzidine-peroxidase (DAB) visualization system (EnVision+, Dako, Carpinteria, CA) or a Bond Polymer Refine Red Detection Kit (Leica Biosystems, Buffalo Grove, IL). Mayer’s hematoxylin was used for counterstaining.

**Imaging and quantification**

For brightfield DIC images, a Zeiss Axio Imager.Z1 compound microscope equipped with an AxioCam HRc was used. For live imaging, zebrafish were anaesthetized using 0.016% tricaine (Sigma) and mounted in 3% methylcellulose (Sigma). A Nikon SMZ1500 microscope equipped with a Nikon digital sight DS-U1 camera was used for capturing both the brightfield and fluorescent images from live zebrafish. For melanoma quantification, all animals in the same experiments were imaged under the same conditions, and the acquired fluorescent images were quantified using the ImageJ software by measuring the pigment or EGFP fluorescence. The pigment or fluorescent area was normalized against the surface area of the fish head to control for varying size of fish. Overlays were created using ImageJ and Adobe Photoshop 7.0.1.

**Drug treatment**

After 2 days of post-transplantation recovery, the 3-week-old juvenile *rag2^-/-^* fish transplanted with *nf1/pten*-mutant melanoma cells were randomly separated and treated with trametinib (Selleck Chemicals, Houston, TX), cobimetinib (Selleck), buparlisib (Selleck), apitolisib (Selleck), sirolimus (rapamycin, LC Laboratories, Woburn, MA), everolimus (LC Laboratories), temsirolimus (LC Laboratories), sorafenib (LC laboratories), sabutoclax (Selleck), obatoclax (Santa Cruz), chloroquine (Sigma), olaparib (Selleck), venetoclax (Chemietek, Indianapolis, IN) and S63845 (Chemgood, Glen Allen, VA) with refreshment every 2 days. Sample size was estimated according to <https://www.statstodo.com/SSizSurvival_Pgm.php>, that with at least 10 animal per group, we will have 90% power to identify 20% tumor suppression, or 95% power to identify 50% tumor suppression, testing at the 0.05 one sided level using a log-rank test. The drug treatment experiments were all blinded that the drug administration and tumor progression monitoring were performed by independent investigators. The treatment conditions were unblinded after the completion of tumor monitoring. Each experiment was replicated at least three times in the laboratory.

**Western blots and antibodies**

Whole-cell lysates were prepared in RIPA buffer. Protein concentration was quantified with a Pierce BCA Protein Assay Kit (Thermo Fisher Scientific Inc.). Equivalent amounts of protein were diluted in the Laemmli samples buffer (Bio-Rad Laboratories) and separated by SDS-PAGE. Proteins were transferred to PVDF membranes (Millipore, Billerica, MA) and subjected to immune blot analysis with each of the specific antibodies for NF1 (Bethyl Laboratories #A300-140A-M; 1:2000 dilution), PTEN (Cell Signaling #9188; 1:1000 dilution), total ERK1/2 (Cell Signaling #4695; 1:1000 dilution), and cleaved caspase 3 (Cell Signaling #9661; 1:500 dilution). All primary antibodies were diluted in 5% milk in PBST (0.5% Tween-20 in PBS).
